# A phylogenetic and taxonomic review of baviine jumping spiders (Araneae, Salticidae, Baviini)

**DOI:** 10.3897/zookeys.1004.57526

**Published:** 2020-12-16

**Authors:** Wayne P. Maddison, Imara Beattie, Kiran Marathe, Paul Y. C. Ng, Nilani Kanesharatnam, Suresh P. Benjamin, Krushnamegh Kunte

**Affiliations:** 1 Departments of Zoology and Botany and Beaty Biodiversity Museum, University of British Columbia, 6270 University Boulevard, Vancouver, British Columbia, V6T 1Z4, Canada; 2 Department of Zoology, University of British Columbia, 6270 University Boulevard, Vancouver, British Columbia, V6T 1Z4, Canada; 3 National Centre for Biological Sciences, Tata Institute of Fundamental Research, GKVK Campus, Bellary Road, Bengaluru 560065, India; 4 205 River Valley Road, #16-53, Singapore 238274, Republic of Singapore; 5 National Institute of Fundamental Studies, Hantana Road, Kandy, Sri Lanka; 6 Department of Zoology, Faculty of Science, Eastern University, Vantharumoolai, Sri Lanka

**Keywords:** Classification, molecular phylogeny, new genus, new species, Salticidae, Salticoida

## Abstract

The systematics and taxonomy of the tropical Asian jumping spiders of the tribe Baviini is reviewed, with a molecular phylogenetic study (UCE sequence capture, traditional Sanger sequencing) guiding a reclassification of the group’s genera. The well-studied members of the group are placed into six genera: *Bavia* Simon, 1877, *Indopadilla* Caleb & Sankaran, 2019, *Padillothorax* Simon, 1901, *Piranthus* Thorell, 1895, *Stagetillus* Simon, 1885, and one new genus, *Maripanthus* Maddison, **gen. nov.** The identity of *Padillothorax* is clarified, and *Bavirecta* Kanesharatnam & Benjamin, 2018 synonymized with it. *Hyctiota* Strand, 1911 is synonymized with *Stagetillus*. The molecular phylogeny divides the baviines into three clades, the *Piranthus* clade with a long embolus (*Piranthus*, *Maripanthus*), the genus *Padillothorax* with a flat body and short embolus, and the *Bavia* clade with a higher body and (usually) short embolus (remaining genera). In general, morphological synapomorphies support or extend the molecularly delimited groups. Eighteen new species are described: *Bavia nessagyna*, *Indopadilla bamilin*, *I. kodagura*, *I. nesinor*, *I. redunca*, *I. redynis*, *I. sabivia*, *I. vimedaba*, *Maripanthus draconis* (type species of *Maripanthus*), *M. jubatus*, *M. reinholdae*, *Padillothorax badut*, *P. mulu*, *Piranthus api*, *P. bakau*, *P. kohi*, *P. mandai*, and *Stagetillus irri*, all **sp. nov.**, with taxonomic authority W. Maddison. The distinctions between baviines and the astioid *Nungia* Żabka, 1985 are reviewed, leading to four species being moved into *Nungia* from *Bavia* and other genera. Fifteen new combinations are established: *Bavia maurerae* (Freudenschuss & Seiter, 2016), *Indopadilla annamita* (Simon, 1903), *I. kahariana* (Prószyński & Deeleman-Reinhold, 2013), *I. sonsorol* (Berry, Beatty & Prószyński, 1997), *I. suhartoi* (Prószyński & Deeleman-Reinhold, 2013), *Maripanthus menghaiensis* (Cao & Li, 2016), *M. smedleyi* (Reimoser, 1929), *Nungia hatamensis* (Thorell, 1881), *N. modesta* (Keyserling, 1883), *N. papakula* (Strand, 1911), *N. xiaolonghaensis* (Cao & Li, 2016), *Padillothorax casteti* (Simon, 1900), *P. exilis* (Cao & Li, 2016), *P. flavopunctus* (Kanesharatnam & Benjamin, 2018), *Stagetillus banda* (Strand, 1911), all **comb. nov.** One combination is restored, *Bavia capistrata* (C. L. Koch, 1846). Five of these new or restored combinations correct previous errors of placing species in genera that have superficially similar palps but extremely different body forms, in fact belonging in distantly related tribes, emphasizing that the general shape of male palps should be used with caution in determining relationships. A little-studied genus, *Padillothorus* Prószyński, 2018, is tentatively assigned to the Baviini. *Ligdus* Thorell, 1895 is assigned to the Ballini.

## Introduction

Baviines are tropical Asian jumping spiders with elongate medium to large bodies, living above ground on leaves or branches of vegetation or in suspended litter (Maddison 2015a). The Baviini is by far the smallest of the four major groups that compose the clade Salticoida (Maddison, 2015a), with far fewer described species (31 to date) than the other three groups (Astioida, ~ 600 species; Marpissoida, ~ 850; Saltafresia, ~ 3500). Baviines have been little studied, but recent works have begun to add to our knowledge quickly (Caleb and Sanap 2017; Kanesharatnam and Benjamin 2018; Malamel et al. 2019; Caleb et al. 2019; Nafin et al. 2020), resulting so far in at least six genera currently placed in the Baviini (*Bavia* Simon, 1877, *Bavirecta* Kanesharatnam & Benjamin, 2018, *Indopadilla* Caleb & Sankaran, 2019, *Padillothorax* Simon, 1901, *Piranthus* Thorell, 1895, and *Stagetillus* Simon, 1885; Maddison 2015a; Kanesharatnam and Benjamin 2018; Malamel et al. 2015, 2019; Caleb et al. 2019). The frequency of new species in recent collecting suggests that the group is much more diverse than it appears; e.g., *Piranthus* includes only two South Asian species described, but there are at least three new species in Singapore alone. The purpose of this paper is to describe some of the recently discovered species, and to present the first phylogeny for the group. The phylogenetic work leads to a revised classification of species into genera, one of which is new.

## Materials and methods

### Material examined

Spider specimens examined for this study are stored in the University of British Columbia Spencer Entomological Collection, Canada (**UBCZ**), the Lee Kong Chian Natural History Museum, Singapore (**LKCNHM**, https://lkcnhm.nus.edu.sg), the Research Collections at National Centre for Biological Sciences, Bengaluru, Karnataka, India (**NCBS**, http://collections.ncbs.res.in), the Florida State Collection of Arthropods (**FSCA**), and the Senckenberg Museum, Frankfurt, Germany (**SMF**). Work on Indian material was done at the National Centre for Biological Sciences, Bengaluru. Work on Sri Lankan material was done at the National Institute of Fundamental Studies, Kandy. Data for specimens collected during fieldwork by WPM and colleagues includes a code beginning “WPM#” representing not a specimen identification, but a collecting event of location and time.

### Morphology

Preserved specimens were examined under both dissecting microscopes and a compound microscope with reflected light. Drawings were made with a drawing tube on a Nikon ME600L compound microscope. Most photographs of living specimens were made with either a Pentax Optio 33WR digital camera with a small lens glued to it for macro capability (2016 and earlier) or an Olympus OM-D E-M10 II camera with 60 mm macro lens (2017 and later). Microscope photographs were made either on a Nikon ME600L compound microscope or an Olympus SZX12 stereoscope and focus stacked using Helicon Focus 4.2.7.

All measurements are given in millimeters. Descriptions of color pattern are based on the alcohol-preserved specimen. Carapace length was measured from the base of the anterior median eyes not including the lenses to the rear margin of the carapace medially; abdomen length to the end of the anal tubercle. The following abbreviations are used:

**ALE** anterior lateral eyes;

**ECP** epigynal coupling pocket (also known as a hood or notch);

**PLE** posterior lateral eyes;

**PME** posterior median eyes (the “small eyes”);

**RTA** retrolateral tibial apophysis;

**TmA** terminal apophysis.

### Molecular data

Molecular data was gathered for three gene regions by traditional Sanger PCR methods, and for many genes by Ultra-Conserved Element (UCE) target enrichment sequencing methods (Faircloth 2017), combined with data from the literature, to assemble a dataset of nine outgroup species and 22 baviine species. Table 1 lists the specimens used in the molecular study; Table 2 lists the data gathered for each. Table 3 lists specimens used for a small auxiliary study of *Nungia* Żabka, 1985 and *Capeyorkia* Richardson, 2016.

**Table 1.** Specimens from which molecular data were used in phylogenetic analysis of baviines.

Species	Specimen ID	Sex	Locality	Lat-Long
** Amycoida **				
*Attulus floricola* (C. L. Koch, 1837)	d545	♂	Poland: Narew	52.9, 23.5
	d030	♂	Canada: Nova Scotia	44.4318, -64.6075
*Breda bicruciata* (Mello-Leitão, 1943)	d471	♀	Uruguay: Lavalleja	-34.426, -55.195
*Colonus hesperus* (Richman & Vetter, 2004)	d472	♂	U.S.A.: Arizona	34.5847, -112.5707
** Astioida **				
*Helpis minitabunda* (L. Koch, 1880)	NZ19-9152	♀	New Zealand	-40.994, 172.994
	S194, S195		New Zealand	
*Ligurra latidens* (Doleschall, 1859)	AS19.3412	♂	Singapore	1.4438, 103.7334
	d175		Singapore	
** Marpissoida **				
*Afromarengo* sp.	MRB262		Gabon	
*Phidippus johnsoni* (Peckham & Peckham, 1883)	d549	♂	Canada: Iona Beach	49.222, -123.216
** Saltafresia **				
*Menemerus bivittatus* (Dufour, 1831)	d559	♀	Singapore	1.4438, 103.7334
	S13/S225		Ecuador	
*Salticus scenicus* (Clerck, 1757)	NA19-2676	♀	Canada: Iona Beach	49.222, -123.216
	d003, S107		U.S.A.	
*** Baviines ***				
*Bavia aericeps* Simon, 1877	2008PNG-2407	♂	Papua New Guinea	-5.231, 142.532
Bavia cf. intermedia (Karsch, 1880)	d079	♂	Malaysia: Sabah: Poring Hot Springs	
*Bavia nessagyna*, sp. nov.	SWK12-4087	♀	Malaysia: Lambir Hills	4.20, 114.037
*Bavia sexpunctata* (Doleschall, 1859)	AS19.2183	♀	Singapore	1.36, 103.77
*Indopadilla bamilin*, sp. nov.	SWK12-1618	♂	Malaysia: Mulu	4.06, 114.829
*Indopadilla kahariana* (Prószyński & Deeleman-Reinhold, 2013)	SWK12-1163	♂	Malaysia: Mulu	4.047, 114.825
	SWK12-1876	♀	Malaysia: Mulu	4.041, 114.817
*Indopadilla kodagura*, sp. nov.	AS19.4314	♂	India: Kodagu	12.22, 75.66
*Indopadilla nesinor*, sp. nov.	MRB076	♀	Singapore	1.39, 103.81
*Indopadilla redunca*, sp. nov.	SWK12-1831	♀	Malaysia: Mulu	4.040, 114.815
*Indopadilla redynis*, sp. nov.	SWK12-0080	♀	Malaysia: Kubah	1.61, 110.19
*Indopadilla sabivia*, sp. nov.	d107	♂	Malaysia: Sabah: Kiabau	5.832, 117.225
*Indopadilla vimedaba*, sp. nov.	SWK12-3620	♀	Malaysia: Mulu	4.042, 114.814
*Stagetillus irri*, sp. nov.	S202	♀	Philippines: Luzon	
Stagetillus cf. opaciceps Simon, 1885	MRB079	♀	Malaysia: Ulu Gombak	3.325, 101.753
*Padillothorax badut*, sp. nov.	d548	♀	Malaysia: Lambir Hills	4.200, 114.035
*Padillothorax flavopunctus* (Kanesharatnam & Benjamin, 2018)	IFS_SAL_1017	j	Sri Lanka	7.2833, 80.6303
Padillothorax cf. flavopunctus	IFS_SAL_679	♀	Sri Lanka	7.3611, 80.8333
*Padillothorax mulu*, sp. nov.	SWK12-2556	♂	Malaysia: Mulu	4.049, 114.86
*Maripanthus draconis*, sp. nov.	d547	♀	Singapore	1.36, 103.77
	d176	♂	Malaysia: Genting Highlands	3.400, 101.777
*Maripanthus reinholdae*, sp. nov.	SWK12-1991	♀	Malaysia: Mulu	4.023, 114.813
	SWK12-1934	♀	Malaysia: Mulu	4.04, 114.817
*Piranthus bakau*, sp. nov.	d424	♂	Malaysia: Bako	1.722, 110.446
Piranthus cf. kohi, sp. nov.	MRB109	j	Malaysia: Ulu Gombak	3.325, 101.753
*Piranthus planolancis* Malamel, Nafin, Sudhikumar & Sebastian, 2019	AS19.5940	♀	India: Mysuru	12.223, 76.627
	AS19.5970	♂	India: Mysuru	12.223, 76.627

**Table 2.** Molecular data used for phylogenetics of baviines. UCE probeset was either arachnid (A) or spider (S). SRA indicates sequence read archive accession number. “Reads Pass QC” indicates number of reads retained after quality control and adapter removal via Illumiprocessor. “UCE loci” counts number of loci both as obtained by mixed spider-arachnid file, and after filtering by occupancy and branch length criteria. “Filtered length” is number of non-missing sites, i.e. total base pairs or sequence length. For 28S, mtDNA, 16SND1, and COI, numbers indicate sequence length as obtained by Sanger sequencing (**σ**) or bycatch from UCE sequencing (**β**). New Sanger sequences show Genbank accession numbers of the form MW0818##. mtDNA indicates taxa for which entire mitochondrial genome recovered as bycatch. Published sequences indicated by citation (MH03: Maddison and Hedin 2003; MN06: Maddison and Needham 2006; MBN8 Maddison, Bodner and Needham 2008; BM12: Bodner and Maddison 2012; ML14: Maddison et al. 2014; MMDH20: Maddison et al. 2020). UCE data for *Breda* and *Colonus* from Maddison et al. 2020a.

Species	Specimen ID	UCE Probes	SRA	Reads pass QC	Contigs	UCE loci	filtered UCE loci	filtered UCE length	28S	mtDNA	167SND1	COI
*Attulus floricola*	d545	A	SRR12832808	4519623	40309	468	129	78353	4797–β			
	d030										770 –σ (MN06)	972–σ (MN06)
*Breda bicruciata*	d471	A	SRX7739885	(MMDH20)	38487	399	87	35038	6720–β	14305–β		
*Colonus hesperus*	d472	A	SRX7739887	(MMDH20)	56975	331	78	28801	6688–β	12586–β		
*Helpis minitabunda*	NZ19-9152	S	SRR12832807	4037736	210843	1096	1031	757228	6751–β	15601–β		
	S194, S195										956–σ (MH03)	1047–σ (MH03)
*Ligurra latidens*	AS19.3412	S	SRR12832798	3565247	25016	1118	1076	760567	5415–β			
	d175										909–σ (BM12)	
*Afromarengo sp.*	MRB262								1049–σ (BM12)		730–σ (BM12)	946–σ (BM12)
*Phidippus johnsoni*	d549	S	SRR12832797	2954316	133630	1103	1038	779778	6734–β	14319–β		
*Menemerus bivittatus*	d559	S	SRR12832796	5327638	74123	1203	1136	770717	5639–β			
	S13/S225										956–σ (MH03)	956–σ (MH03)
*Salticus scenicus*	NA19-2676	A	SRR12832795	2854670	25200	462	122	73552	6651–β			
	d003, S107										909–σ (MH03)	972–σ (BM12)
*** Baviines ***												
*Bavia aericeps*	2008PNG-2407	S	SRR12832794	3940528	189254	1225	1162	912384	6782–β	14324–β		
*Bavia cf. intermedia*	d079								754–σ (MBN8)		854–σ (ML14)	972–σ (MBN8)
*Bavia nessagyna*	SWK12-4087	S	SRR12832793	497179	5132	894	860	410659	2772–β			
*Bavia sexpunctata*	AS19.2183	S	SRR12832792	1638329	77954	1236	1179	911457	5677–β	14346–β		
*Indopadilla bamilin*	SWK12-1618										687–σ MW081881	
*Indopadilla kahariana*	SWK12-1163	S	SRR12832791	2410712	16408	1187	1145	820455	4759–β		675–σ MW081883	596–β
	SWK12-1876								1042–σ MW081869		632–σ MW081882	
*Indopadilla kodagura*	AS19.4314	S	SRR12832806	542868	4171	895	863	525638	6758–β		1136–β	1083–β
*Indopadilla nesinor*	MRB076										920–σ MW081884	960–σ MW081865
*Indopadilla redunca*	SWK12-1831								1061–σ MW081871		650–σ MW081885	
*Indopadilla redynis*	SWK12-0080	S	SRR12832805	307232	4791	917	879	483961	435–β			
*Indopadilla sabivia*	d107								771–σ MW081872			952–σ MW081866
*Indopadilla vimedaba*	SWK12-3620										608–σ MW081886	
*Stagetillus irri*	S202								656–σ (MH03)		963–σ (MH03)	969–σ (MH03)
*Stagetillus cf. opaciceps*	MRB079	S	SRR12832804	1033936	9649	1077	1047	772184	6894–β		967-βσ (MBN8,β)	467–β
*Padillothorax badut*	d548	S	SRR12832803	3504035	217449	1238	1175	906962	5682–β	14231–β		
*Padillothorax flavopunctus*	IFS_SAL_1017								729–σ MW081874			557–σ MW081867
*Padillothorax cf. flavopunctus*	IFS_SAL_679								695–σ MW081875			544–σ MW081868
*Padillothorax mulu*	SWK12-2556								1033–σ MW081873		659–σ MW081887	
*Maripanthus draconis*	d547	S	SRR12832802	2632437	127899	1207	1159	881761	5622–β	14399–β		
	d176								821–σ MW081878			
*Maripanthus reinholdae*	SWK12-1991	S	SRR12832801	1554841	72255	1170	1139	890576	5243–β		1653–β	
	SWK12-1934								1043–σ MW081877			
*Piranthus bakau*	d424								1044–σ MW081879		720–σ MW081888	
*Piranthus cf. kohi*	MRB109								1067–σ MW081880			
*Piranthus planolancis*	AS19.5940	S	SRR12832800	3050561	135553	1217	1173	924020	6749–β	14872–β		
	AS19.5970	S	SRR12832799	1235845	56562	1186	1157	895555	6749–β	14683–β		

**Table 3.** *Nungia* and *Capeyorkia* specimens sequenced for phylogenetic study, with Genbank accession numbers.

Species	Specimen ID	Sex	Locality	Lat-Long	28S	16SND1
*Nungia hatamensis* (Thorell, 1881)	d260	♂	Papua New Guinea: Putuwé	-5.231, 142.532		MW202326
*Nungia xiaolonghaensis* (Cao & Li, 2016)	MRB078	♂	Malaysia: Tanah Rata	4.46, 101.40	MW187118	MW202327
*Nungia* sp. “NPNGK”	d259	♂	Papua New Guinea: Tualapa	-5.283, 142.498	MW187119	
*Nungia* sp. “NSGPQ”	d178	♂	Singapore	1.44, 103.70	MW187120	MW202328
*Nungia* sp. “NUBWH”	SWK12-3204	♀	Malaysia: Mulu NP	4.042, 114.814	MW187121	MW202329
*Nungia* sp. “NUMUL”	SWK12-1943	♂	Malaysia: Mulu NP	4.0405, 114.817	MW187122	MW202330
Capeyorkia cf. vulpecula (Thorell, 1881)	MRB087	♂	Papua New Guinea: Bundun	-6.8600, 146.6178	MW187123	
*Capeyorkia* sp. “NPNGE”	d261	♂	Papua New Guinea: Varirata NP	-6.07, 145.40		MW202331
*Capeyorkia* sp. “NPNGF”	d258	♂	Papua New Guinea: Goroka	-9.436, 147.364	MW187124	MW202332

#### UCE Data

DNA was isolated using the Qiagen DNeasy Blood and Tissue Kit, following the spin-column protocol. Quality of the isolation was estimated using a NanoDrop 2000c Spectrophotometer, and samples were repeated where possible if the 260:280 nm UV absorbance ratio fell outside the range of 1.4 to 2.2. For most taxa 1 to 4 legs were used for DNA extraction, but the entire prosoma was used for *Padillothorax badut* (specimen d548) and *Helpis minitabunda* (specimen NZ19-9152). For the target enrichment UCE sequencing, dual-indexed TruSeq-style libraries were prepared following methods previously used in arachnids (e.g., Starrett et al. 2017; Derkarabetian et al. 2018; Hedin et al. 2018; Kulkarni et al. 2019). Targeted enrichment was performed using either the myBaits Arachnida 1.1Kv1 (Arbor Biosciences; Faircloth 2017; Starrett et al. 2017) or the Spider 2Kv1 kit (Arbor Biosciences; Kulkarni et al. 2019) following the myBaits v4.01 protocol (https://arborbiosci.com/wp-content/uploads/2018/04/myBaits-Manual-v4.pdf). Libraries were sequenced on partial lanes of Illumina NovaSeq 6000 S4 runs with 150 bp paired end reads. To the resulting set of reads we added those from two amycoid taxa, *Breda* and *Colonus*, obtained by Maddison et al. (2020), to assist as outgroups. Raw demultiplexed reads were processed with Phyluce version 1.6 (Faircloth 2016), quality control and adapter removal were conducted with the Illumiprocessor wrapper (Faircloth 2013), and assemblies were created with SPAdes version 3.14.1 (Nurk et al. 2013), using the meta option, at default settings.

From among the contigs thus assembled, those matching particular UCE probes were pulled out using the Phyluce pipeline at default settings. Because some taxa were captured using the arachnid probeset (outgroups *Attulus*, *Breda*, *Colonus*, *Salticus*), and others using the spider probeset (remaining outgroups, and all baviines), a blended probeset file was needed to best pull out UCE contigs, because each of the arachnid and spider probesets includes loci not included by the other. Kulkarni et al.’s (2019) spider probeset includes (i) some of Starrett et al.’s (2017) arachnid probes directly, (ii) others for the same loci but modified to target spiders better, and (iii) others for new loci. Because Kulkarni et al. do not identify probes of the second category as such, we sought to identify whether spider probes are orthologous to arachnid probes. We then deleted from the probeset file those arachnid probes matching spider probes, as including duplicate homologs reduces data recovery (contigs matching two probes are removed by Phyluce for being problematical). To determine homology, contigs from 18 diverse species in the Salticinae, 12 captured with arachnid probes, 6 with spider probes, were each matched against both arachnid and spider probesets. Any instance of a contig matching both a spider probe and arachnid probe, as assessed using a script examining .lastz files, was taken as indicating homology between the probes. Arachnid probes that showed no such hint of homology to spider probes were then added to Kulkarni et al.’s (2019) spider probeset to generate the blended probeset (see Suppl. material 1). The spider probeset includes 15015 probes and probe parts; the arachnid probeset, 14799; blended, 25689. The efficacy of the blended probeset can be seen in the numbers of loci recovered in the baviine dataset reported here: the arachnid probset pulled out on average 134 loci from spider-enriched taxa and 411 from arachnid-enriched taxa; the spider probeset pulled out on average 1118 and 113 respectively; the blended probeset pulled out on average 1123 and 415. Nonetheless, many of the UCE loci recovered from the arachnid-enriched taxa were only among those taxa; this explains why many were subsequently deleted when a filter for occupancy among ingroups (see below) was applied.

Recovered UCE loci were aligned with MAFFT (Katoh and Standley 2013) and trimmed with Gblocks (Castresana 2000; Talavera and Castresana 2007), using –b1 0.5, –b2 0.5, –b3 10, –b4 4 settings in the Phyluce pipeline. Among the loci recovered, those with fewer than 6 taxa total or fewer than 3 ingroups were deleted. As in the analysis of Maddison et al. (2020), loci were also deleted over concerns about paralogy if their gene tree showed a very long branch, at least 5× longer than the second longest branch.

#### Data for 28S and mitochondrial genes

For the new Sanger-sequenced data, specimens were preserved, their DNA extracted, and sequences obtained for the nuclear gene 28S and the mitochondrial gene regions 16SNDI and COI following the protocols using the protocols of Zhang and Maddison (2013) and Maddison et al. (2014). Alignments were done by MAFFT with the L-INS-i option.

The same three gene regions were also present among the sequence capture genomic contigs as untargeted bycatch. We recovered them by constructing a local BLAST database of the contigs of each taxon, and querying it with 28S, 16SND1 and COI sequences from eight to nine different salticid species (*Bavia*, *Indopadilla*, *Bathippus*, *Harmochirus*, *Idastrandia*, *Langerra*, *Phintella*, *Platycryptus*, *Salticus*, *Attulus*, *Lyssomanes*), retaining any contigs matching with an e-value of less than 10^-10^ and length greater than 200. In a few cases, multiple contigs from a taxon were recovered as matching a single locus, but after alignment against others these could be interpreted as different parts of the target gene, and were thus stitched together to a single sequence.

### Phylogenetic analysis

Maximum likelihood phylogenetic analyses were performed with IQ-TREE version 1.6.7.1 (Nguyen et al. 2015) using the Zephyr 3.1 package (Maddison and Maddison 2020) in Mesquite 3.61 (Maddison and Maddison 2019) on several datasets derived from the UCE, bycatch, and Sanger-sequenced data. The datasets were:

1. “UCEs” – The UCE dataset after filtering of loci, concatenated, unpartitioned.
2. “mtDNA+28S” – The concatenated data from 28S and mitochondrial sequences (10 full mtDNA, others Sanger and bycatch 16SND1 aligned against the full mtDNA). Analyzed with 2 partitions, 28S and mtDNA.
3. “restricted mtDNA+28S” – A restricted version of 28S and mtDNA with the long bycatch sequences trimmed to put the taxa with legacy Sanger data on almost equal footing (i.e., approximately as much data) with those with bycatch. 28S sites at start and end were trimmed until at least 3 of the shorter legacy Sanger sequences were represented. The same rule for trimming was used for the mtDNA before 16SND1, between 16SND1 and CO1 genes, and after COI. Analyzed with 2 partitions, 28S and mtDNA.
4. “UCEs+mtDNA+28S” – The UCE loci (dataset #1) concatenated to the restricted 28S and mtDNA data (dataset #3). Analyzed unpartitioned.

For the partitioned analyses, the options -m MFP -spp were used (extended model selection followed by tree inference, edge-linked partition model, with partition-specific rates); for the unpartitioned analyses, -m MFP (extended model selection followed by tree inference, edge-linked partition model, no partition-specific rates).

Several species of elongate brown Asian and Australasian salticids were initially identified in the field as baviines, but were excluded from the Baviini by preliminary molecular analyses and closer morphological study, which showed them to be near *Nungia epigynalis* Żabka, 1985. To document this and clarify the limits of the Baviini, we did a small analysis based on 28S and 16SND1, using previously published sequences of baviines, astioids, and other groups from Maddison and Hedin (2003), Bodner and Maddison (2012), and Maddison et al. (2014) combined with new sequences of the species in question (Table 3). Details are given in the Taxonomy section under Viciriini: *Nungia*.

Raw sequence reads from UCE capture are deposited in the Sequence Read Archive (BioProject PRJNA667925, https://www.ncbi.nlm.nih.gov/sra/PRJNA667925) with accession numbers shown in Table 2. Genbank accession numbers of Sanger sequenced genes are shown in Tables 2 and 3. Alignments and trees are deposited in the Dryad data repository (https://doi.org/10.5061/dryad.4f4qrfj9j).

### Taxonomic authority

The taxonomic authority for all nomenclatural acts (synonymies, new combinations, new species) is W. Maddison.

## Molecular phylogenetic results

### Molecular data obtained

UCE data obtained are outlined in Table 2. The Phyluce pipeline recovered aligned matrices of 1837 loci using the blended spider-arachnid probeset. In these initially recovered loci, the 16 taxa originally sequence-captured with the spider probeset had on average 1123 loci and 797,222 base pairs of sequence; the four originally sequence-captured with the arachnid set had on average 415 loci and 158,475 base pairs. From the original 1837 loci, 511 were deleted because they had fewer than six taxa total or fewer than three ingroups. Thirteen loci were deleted for having the longest branch more than 5 × longer than the second longest. This resulted in resulting in a final set of 1313 loci, in which the 16 spider-probeset taxa had on average 1076 loci and 775,244 base pairs of sequence; the four arachnid-probeset taxa had on average 104 loci and 53,936 base pairs. The strong decline in arachnid-probeset taxa from 415 loci to 104 after occupancy filtering suggests that Kulkarni et al.’s (2019) incorporation of arachnid-unique probes into the spider probeset included only a portion. The 1313 loci were concatenated into a single alignment with 1,050,217 sites.

The bycatch 28S sequences were 435–6894 bp long (average 5675.9) and aligned well with the Sanger 28S sequences using MAFFT at default settings. Bycatch 28S were obtained for three specimens for which previous Sanger sequences were available and identical (*I. kahariana* SWK12-1163, 1021 base pairs; *M. reinholdae* SWK-01991, 1037 bp; S. cf. opaciceps MRB079, 1067 bp) except for a one base difference at the start of the *I. kahariana* sequence. An initial alignment of 28S including UCE bycatch and legacy Sanger data was 18646 bp long, but the first 1593 bp and the last 9793 bp of the alignment were poorly aligned and represented by only a few bycatch sequences, and so were trimmed. After addition of a few other taxa and realignment, the final 28S alignment was 7281 bp long.

Among the bycatch contigs for 10 taxa were long sequences containing both the 16SND1 and COI regions, and whose size (12568–15601 bp) suggests they may be the whole or nearly whole mitochondrial genome (marked in the column “mtDNA” in Table 2). For other taxa, the mitochondrial contigs recovered as matching 16SND1 and COI were separate and fairly short (16SND1: 362, 1136, 1835 bp; COI: 467, 596, 1083 bp). Bycatch 16SND1 were obtained for two species for which previous Sanger sequences were available and identical. For S. cf. opaciceps (MRB079) the 362 bp bycatch sequence was identical to the 908 bp Sanger sequence in the 303 bp of overlap; the sequence used was their concatenation. For *Helpis*, the 956 bp Sanger sequence published by Maddison and Hedin (2003) matches exactly the longer bycatch sequence here obtained from a different specimen, NZ19-9152, also from New Zealand. The COI obtained from those same two specimens (Sanger and bycatch likewise, respectively) is identical except for one nucleotide, but the 721 bp sequence reported by Maddison et al. (2014) for a different specimen of *Helpis* from Papua New Guinea differs at 35 sites, and is thus likely a different species. The bycatch sequences were used in their entirety, except for a terminal 183 bp of a bycatch 16SND1 from *M. reinholdae* SWK12-1991, which was deleted because it showed no clear alignment with that portion of other taxa; we suspect that portion may have mistakenly assembled with 16SND1 because of a shared poly-AT repeat.

The whole mtDNA sequences aligned well against each other using MAFFT at default settings. The shorter bycatch sequences and Sanger 16SND1 and COI aligned well against the whole mtDNA. The whole mtDNA sequences initially differed in their (arbitrary) starting point on the circular mitochondrial genome, but after a preliminary alignment they were adjusted to all begin at a conserved region in 16S. After adjustments by hand to align ND1 and COI without gaps, the other regions (before ND1, between ND1 and COI, and between COI and the end) were re-aligned using MAFFT.

Trimming of the 28S and mtDNA alignments as explained for dataset #3 (“restricted mtDNA+28S”) yielded a 28S alignment of 1181 base pairs, and a mitochondrial alignment of 2211 bp.

### Phylogenetic results

**Figures 1–3.**
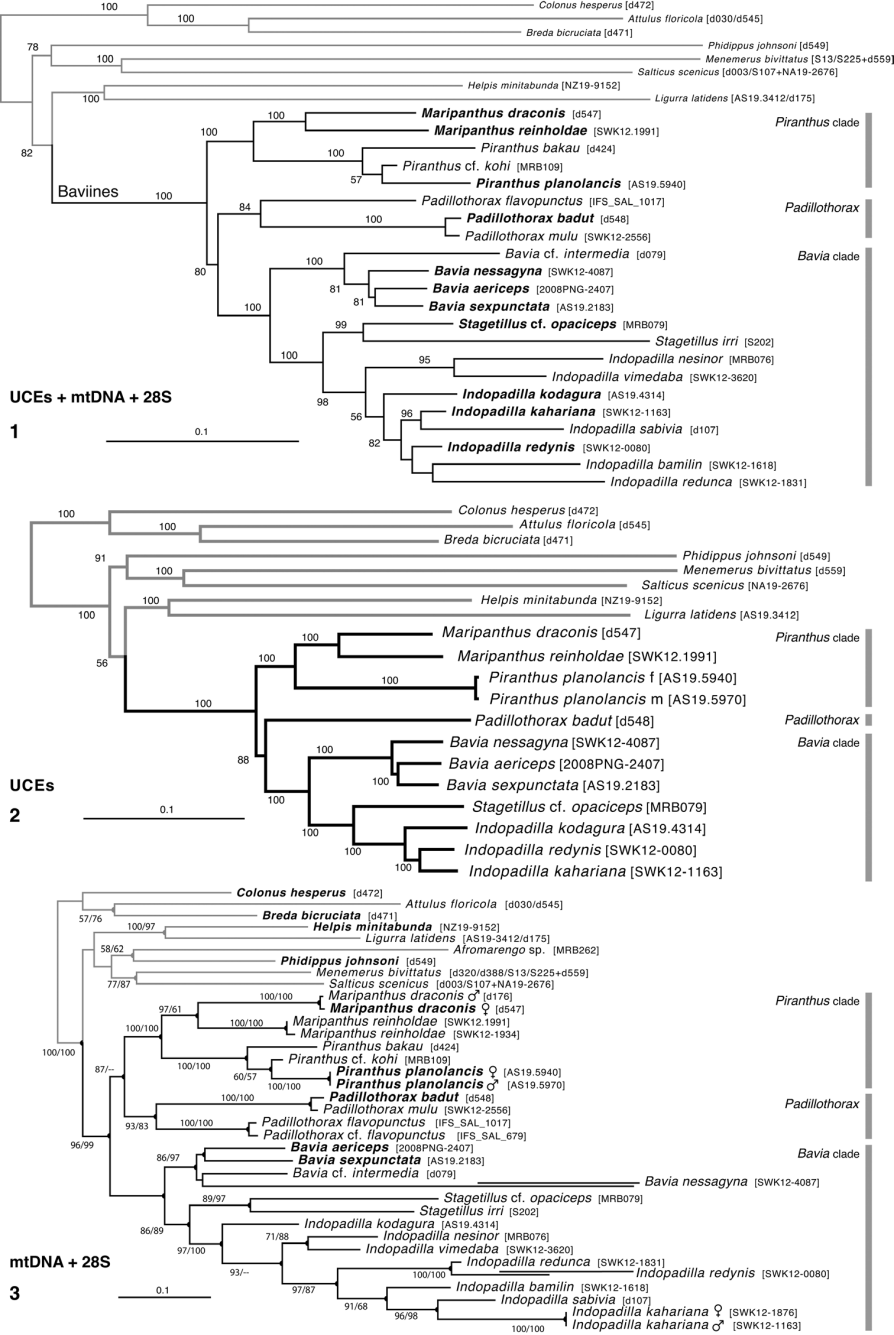
Phylogeny. **1** Maximum Likelihood tree (best of 20 replicates) from combined data set of 1313 UCE loci, plus mitochondrial 16SND1 and COI regions, plus 28S (dataset #4 in Methods). Baviines whose names are in bold, and all outgroups, have UCE data. Numbers are percentage of 500 bootstrap replicates showing the clade **2** Maximum Likelihood tree (best of 50 replicates) from concatenated data from 1313 UCE loci (dataset #1 in Methods). Numbers are percentage of 1000 bootstrap replicates showing the clade **3** Maximum Likelihood tree (best of 50 replicates) from concatenated data from mitochondrial data and 28S (dataset #2 in Methods). Taxa in bold have data from the entire mitochondrial genome, or nearly so. Numbers are percentage of 1000 bootstrap replicates showing the clade for the full dataset (#2), followed by the bootstrap percentage for the restricted mtDNA+28S dataset (#3 in Methods). Spots at nodes show those clades that also appear in the ML tree in the restricted dataset. The branches to *B. nessagyna* and *I. redynis* are long, compacted visually by cutting and sliding part of the length over itself; the actual length therefore should be seen as longer by the length of the overlap.

The primary phylogenetic results are shown in Figs 1–3. The different datasets (UCE, Sanger, and combined) gave substantially concordant results, with just a few points of disagreement. All confirm the monophyly of the baviines, and divide the group into three major clades (marked in Figs 1–3), one with a relatively long embolus that appears freely moveable (the *Piranthus* clade), one with a short erect embolus and flat body (the genus *Padillothorax*), and one with a relatively short embolus largely fixed to the tegulum and a higher body (the *Bavia* clade). The relationship among these three groups varies by dataset: *Padillothorax* is sister to the *Piranthus* clade by the mitochondrial and 28S data (Fig. 3), but by the much larger UCE and combined datasets it is placed reasonably securely as a deep-branching sister to the *Bavia* clade.

Relationships within each of the three major clades is reasonably stable across datasets. Within the *Piranthus* clade, the morphologically distinctive *Piranthus* is monophyletic, as is *Maripanthus*. The morphologically similar *Padillothorax badut* and *P. mulu* are sisters, as are *P. flavopunctus* and P. cf. flavopunctus. Within the *Bavia* clade, molecular results more or less match morphological groups: *Bavia* with relatively large bodies, *Indopadilla* with ridged chelicerae, thoracic bulges, and exposed clypeal arthrodial membrane, and the elongate yellow-orange *Stagetillus*. Accordingly, the concepts of genera here come from both morphological and molecular evidence.

### Taxonomic results

#### Tribe Baviini Simon, 1901

**Genera included**:

*Bavia* Simon, 1877

*Indopadilla* Caleb & Sankaran, 2019

*Maripanthus* Maddison, gen. nov.

*Padillothorax* Simon, 2001

*Padillothorus* Prószyński, 2018

*Piranthus* Thorell, 1895

*Stagetillus* Simon, 1885

Narrow-bodied medium to large salticids in Asia and Australasia, pluridentate, with an embolus fixed to the tegulum or with some degree of mobility. The abdomen is usually long and the legs (except the first) relatively short. There is no known clearly understood morphological synapomorphy of the group. Nonetheless, molecular data groups together most tropical Asian salticids of this body form as baviines. Some long-bodied ballines (e.g., *Mantisatta* Warburton, 1900, *Copocrossa* Simon, 1901), marpissines (*Mendoza* Peckham & Peckham, 1894), astioids (*Holoplatys* Simon, 1885 and relatives, *Nungia* Żabka, 1985), chrysillines (*Epocilla* Thorell, 1887), and plexippines (*Telamonia* Thorell, 1887) might be confused for baviines, but most have distinctive features of their own. Most difficult to distinguish from baviines are perhaps the viciriine astioids *Nungia* and relatives (including *Pungalina* Richardson, 2013 and *Capeyorkia* Richardson, 2016). *Nungia* are generally smaller than baviines, with a more flat-topped carapace; they are quite distinct by molecular data (Maddison et al. 2014, and see below under Viciriini), but the palps are similar. *Nungia* is discussed further under Viciriini, below.

*Padillothorus* (see Prószyński 1984, 2018) is included tentatively among the baviines based primarily on the similarity of its body form with baviines, and its geographic distribution. *Padillothorus* has five retromarginal cheliceral teeth (Reimoser, 1927), which is typical for baviines. Although *Ligdus* Thorell, 1895 might appear as a candidate to belong in the Baviini, with narrow body and raptorial front legs, the juvenile type specimen (in NHM London, examined) is such a close match to *Copocrossa* Simon, 1901 as to be possibly a senior synonym thereof. *Ligdus* shares with *Copocrossa* and *Mantisatta* the peculiar feature of a first metatarsus bent ventrally almost 90 degrees near its base. Because of its apparent relationship with these ballines, *Ligdus* is therefore moved to the Ballini.

Figures 4–35 illustrate character variation in baviines, some of which is used to diagnose genera. Among baviines, the genera vary in these characters:

**Figures 4–35.**
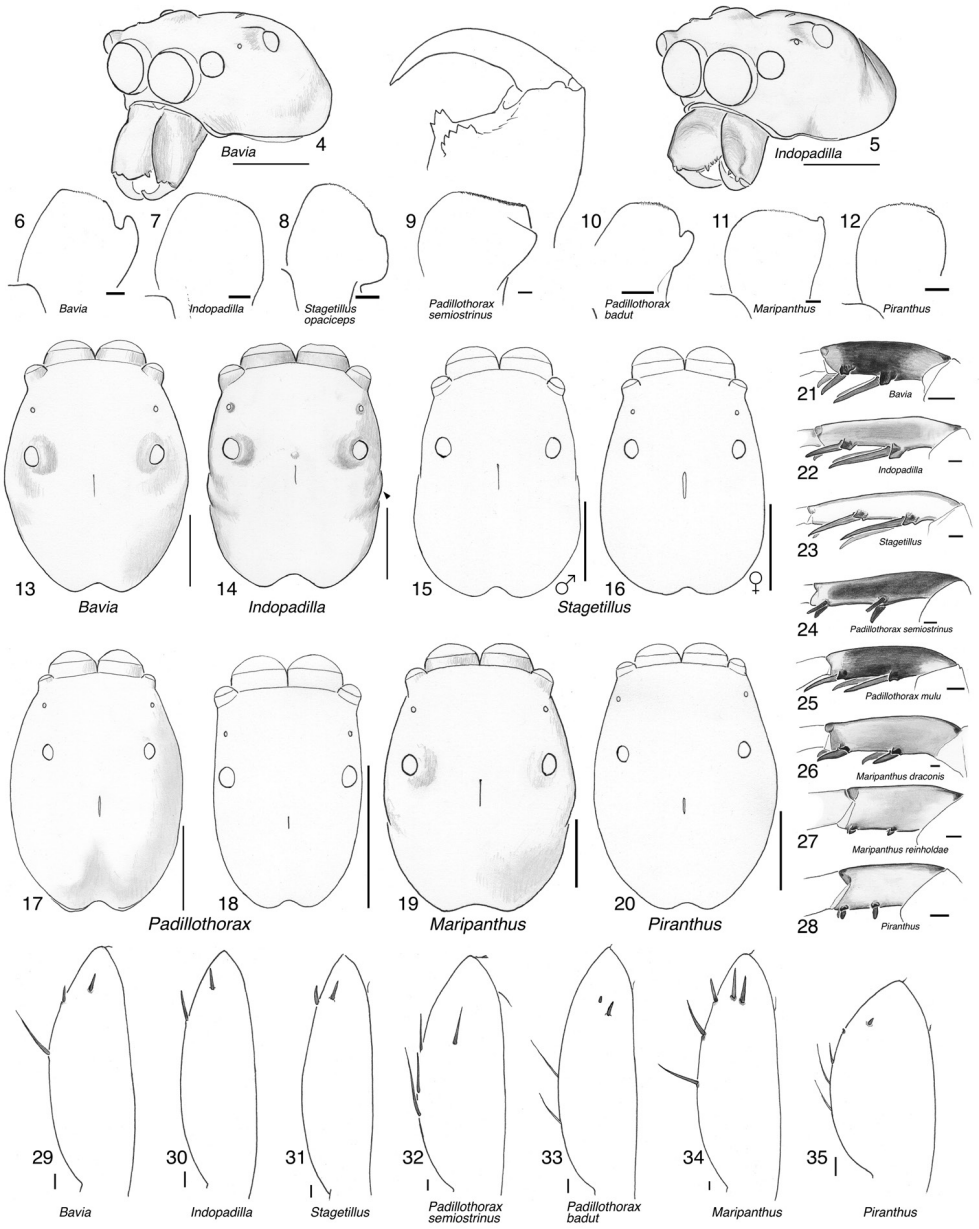
Variation in traits among baviines **4, 5** oblique views of female prosoma **4***Bavia nessagyna* (specimen IDWM.20004; note convex front face of chelicerae, and typical thorax) **5***Indopadilla redunca* (holotype IDWM.20011; note concave front face of chelicerae, and thoracic bulges) **6–12** left male endite (9 also with chelicera) **6***Bavia nessagyna* (holotype IDWM.20005) **7***Indopadilla redunca* (specimen SWK12-M0009) **8***Stagetillus opaciceps* (specimen JK.08.08.19.0001) **9***Padillothorax semiostrinus* (specimen JK.20.06.20.001) **10***Padillothorax badut* (specimen SWK12-4688) **11***Maripanthus jubatus* (specimen AS19.4373) **12***Piranthus planolancis* (specimen AS19.5970) **13–20** carapace (all females except **15**) **13***Bavia nessagyna* (specimen IDWM.20004) **14***Indopadilla redunca* (holotype IDWM.20011) **15***Stagetillus opaciceps* male (specimen JK.13.02.26.0017) **16***Stagetillus* cf. *opaciceps* female (specimen MRB079).**17***Padillothorax semiostrinus* (specimen JK.20.06.20.001) **18***Padillothorax mulu* (from Mulu Nat. Pk.) **19***Maripanthus reinholdae* (specimen SWK12-1934) **20***Piranthus planolancis* (specimen AS19.5940) **21–28** Metatarsus of first leg, retrolateral view. All female except **24** male. All are of left legs except **23, 25, 28** which are of right leg, digitally flipped **21***Bavia nessagyna* (specimen IDWM.20004) **22***Indopadilla kahariana* (specimen SWK12-1876) **23***Stagetillus* cf. *opaciceps* (specimen MRB079) **24***Padillothorax semiostrinus* (specimen SWK12-EP0105) **25***Padillothorax mulu* (specimen SWK12-EP0105) **26***Maripanthus reinholdae* (specimen SWK12-1934) **27***Maripanthus draconis* (from Gunung Belemut, Johor) **28***Piranthus planolancis* (specimen AS19.5940) **29–35** prolateral surface of male first leg femur. **29***Bavia* cf. *capistrata* (specimen from Singapore) **30***Indopadilla kahariana* (specimen from Lambir Hills Nat. Pk.) **31***Stagetillus opaciceps* (specimen JK.08.08.19.0001) **32***Padillothorax semiostrinus* (specimen JK.20.06.20.001) **33***Padillothorax badut* (specimen SWK12-4688) **34***Maripanthus draconis* (specimen from Johor, Gunung Lambak) **35***Piranthus bakau* (holotype). Scale bars: 0.1 mm (**6–12, 21–35**), 1.0 mm (**4, 5, 13–20**).

Shape of the carapace – flatter (height < 36% of length) in most *Piranthus* and *Padillothorax*, higher in others. *Indopadilla* has distinctive bulges on the thorax sides (Figs 5, 14).

Lateral margin of male endites – *Indopadilla* and *Piranthus* males have the endite with a simple rounded margin, but the other genera have varying projections (Figs 6–12). *Maripanthus* bears a sharp distal-retrolateral corner. *Bavia* and *Padillothorax* have a lobe, in some thumb-like (Figs 6, 10), in others broad and triangular (Fig. 9). *Stagetillus opaciceps* has a large broad lobe projecting retrolaterally (Fig. 8), though *S. irri* lacks this lobe, and instead has only a mild corner at the retrolateral distal tip, not too different from *Indopadilla*.

Position of the fovea (Figs 13–20) – well back of the posterior eyes in *Piranthus*, *Padillothorax*, and *Padillothorus* (see p. 95 of Prószyński 1984); just behind the PME in others.

Sockets of leg I macrosetae – in the *Bavia* clade, the sockets of macrosetae extend downward as lateral flange (Figs 21–23), especially notable on the metatarsus, whose macrosetae are at least as long as the metatarsus is wide. In the *Piranthus* clade, sockets and macrosetae are much shorter (Figs 26–28), except in males of *Maripanthus draconis* and *M. jubatus*, which have fairly long macrosetae on the first metatarsus. *Padillothorax* is variable (Figs 24, 25).

Macrosetae of first femur (Figs 29–35) – *Padillothorax* is unusual among salticids in having one or two macrosetae more or less centrally placed on the prolateral face of the first leg femur. Most other salticids have femoral macrosetae, but with few exceptions (in *Epocilla* [Ali et al. 2018: fig. 2], *Padilla* Peckham & Peckham, 1894 [Andriamalala 2007], and some marpissines [Edwards 2006]) they are in a more dorsal or distal position; in *Padillothorax* they are at least one quarter of the femur’s length from the distal tip, approx. midway between dorsal and ventral.

In several baviine genera, not each others’ closest relatives, there is a characteristic series of markings consisting of small patches of pale scales on the thorax: one patch medially between the PLE, a short longitudinal stripe at the top of the thoracic slope, and one behind each PLE (e.g., *Bavia*, Fig. 53; *Indopadilla*, Fig. 84, *Maripanthus*, Fig. 222).

The taxonomic account below presents in sequence the *Bavia* clade, *Padillothorax*, and then the *Piranthus* clade.

#### The *Bavia* Clade (*Bavia*, *Indopadilla*, *Stagetillus*)

##### Bavia

Taxon classificationAnimaliaAraneaeSalticidae

Simon, 1877

249FE9A8-F940-5E45-A836-0E20B9A250C3

Bavia Simon 1877. Type species Bavia aericeps Simon, 1877
Acompse L. Koch 1879. Type species Acompse suavis L. Koch, 1879 = B. aericeps.

###### Species included.

*Bavia aericeps* Simon, 1877

*Bavia capistrata* (C. L. Koch, 1846), combination restored, removed from synonymy with *Evarcha flavocincta* (C. L. Koch, 1846)

*Bavia fedor* Berry, Beatty & Prószyński, 1997

*Bavia nessagyna* Maddison, sp. nov.

*Bavia gabrieli* Barrion, 2000

*Bavia intermedia* (Karsch, 1880)

*Bavia maurerae* (Freudenschuss & Seiter, 2016), comb. nov., transferred from *Epidelaxia*

*Bavia planiceps* (Karsch, 1880)

*Bavia sexpunctata* (Doleschall, 1859)

*Bavia valida* (Keyserling, 1882)

###### Diagnosis.

Larger-bodied than most other baviines. Carapace relatively broad and having hexagonal shape, widest at or just behind the PLEs (Fig. 13). Chelicerae lack the sharp lateral ridge (Fig. 4) of *Indopadilla*. Embolus shorter than length of tegulum, arising in all known species on bulb’s distal prolateral corner. ECP on a prominent medial bulge. Male endite with small thumb-like lobe laterally (Fig. 6), as in the *Padillothorax badut* group.

**Figures 36–41.**
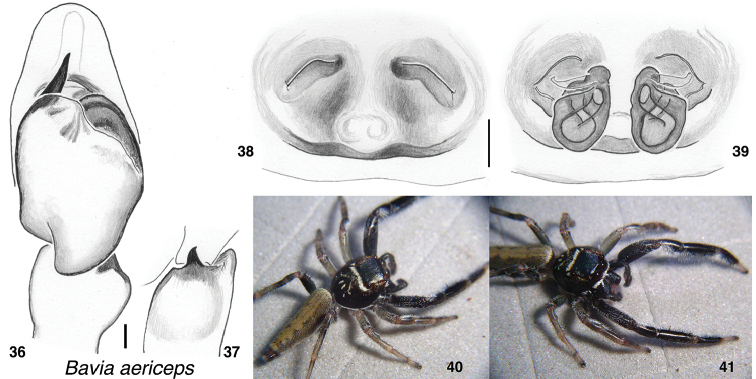
*Bavia aericeps***36** male left palp, ventral view (specimen 2008PNG-2407, Papua New Guinea, 5.231°S, 142.532°E) **37** same, retrolateral view of tibia **38** female epigyne, ventral (specimen SMF 60114, Samoa) **39** same, vulva. **40, 41** male (specimen 2008PNG-1517, Papua New Guinea, 5.283°S, 142.498°E). Distance between substrate grooves 10 mm. Scale bars: on genitalia 0.1 mm.

Illustrations are given here of some of the well-known species of *Bavia*, including *B. aericeps* (Figs 36–41) and *B. sexpunctata* (Figs 54–63). *B. capistrata* was synonymized without explanation by Prószyński (2017) with the extremely different *Evarcha flavocincta*, possibly because of superficial similarities in the palp. C. L. Koch’s (1846) illustration of the male of *Maevia capistrata* is clearly a *Bavia* by body form and markings. The only doubt about the status of C. L. Koch’s species is which species of *Bavia* is it precisely. Candidates include the one figured by Cao, Li, and Żabka (2016), the one figured here as B. cf. capistrata (Figs 42–53), and *B. nessagyna* (Figs 64–75). The one figured here as B. cf. capistrata could be different from that figured by Cao, Li, and Żabka. The former shows a slightly wider embolus and more delicate ECP, and possibly more contrasting markings. Regardless, C. L. Koch’s species is removed from synonymy with *Evarcha flavocincta* and returned to *Bavia*.

**Figures 42–53.**
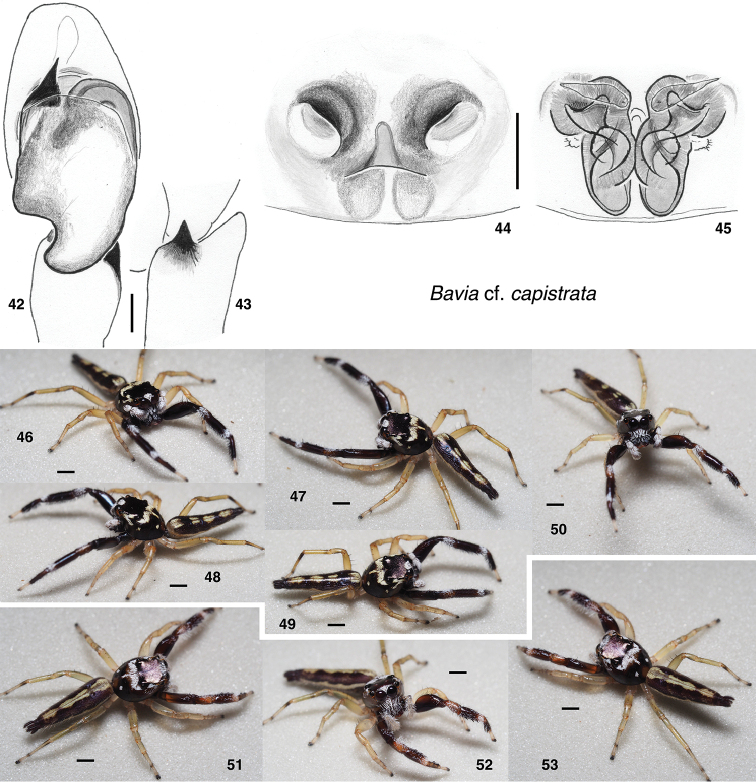
*Bavia* cf. *capistrata*. **42** male left palp, ventral view (specimen AS19.1118, Singapore) **43** same, retrolateral view of tibia **44** epigyne, ventral (specimen AS19.1128, Singapore) **45** vulva, dorsal **46–49** Male AS19.1118 **50** Male AS19.2341 (Singapore) **51–53** female AS19.1128 (Singapore). Scale bars: on genitalia 0.1 mm; on bodies 1.0 mm.

A video of the living female B. cf. capistrata (specimen AS19.1128) is available in Maddison (2020).

In addition to the species below, we have seen an undescribed species near *B. nessagyna* from Mulu National Park (single female) and a species near *B. intermedia* (single male, here represented as specimen d079 in the Sanger data).

##### Bavia nessagyna

Taxon classificationAnimaliaAraneaeSalticidae

Maddison, sp. nov.

4C88D66A-AFC9-50DB-A8DB-96C11646C423

http://zoobank.org/38524691-9D72-4BD7-9EED-76B3E580EA43

Figs 5 , 6 , 13 , 21 , 64–75

###### Type material.

All from Malaysia: Sarawak: Lambir Hills Nat. Pk., and in UBCZ. ***Holotype***: male IDWM.20005 from Bukit Pantu Trail, 4.2032°N, 114.0305°E to 4.2035°N, 114.0304°E, 210 m el. 5 April 2012 Maddison/Piascik/Ang WPM#12-135. ***Paratypes***: Male SWK12-4726 from Inoue Trail, 4.2002°N, 114.0346°N, to 4.2004°N, 114.0342°E, 200 m el. 4 April 2012 Maddison/Piascik/Ang WPM#12-130; female SWK12-4087 from Inoue Trail, 4.1990°N, 114.0375°E to 4.1988°N, 114.0370°E, 120 m el. 1 April 2012 Maddison/Piascik WPM#12-113; female IDWM.20004 from Lepoh-Ridan Trail, 4.2022°N, 114.0279°E to 4.2019°N, 114.0278°E 170 m el. 2 April 2012 Maddison/Piascik WPM#12-124; female IDWM.20006 from Bukit Pantu Trail, 4.2028°N, 114.0305°E to 4.2032°N, 114.0305°E, 210 m el. 5 April 2012 Maddison/Piascik/Ang WPM#12-134; female IDWM.20003 from Pantu Trail, 4.2030°N, 114.0399°E to 4.2032°N, 114.0396°E, 150–160 m el. 6 April 2012 Piascik/Ang WPM#12-145.

###### Etymology.

From the Greek *nessa*, duck, and *gyne*, female, referring to the resemblance of the epigyne to a duck’s bill. *Other names*: In WPM’s lab notebooks the informal code for this species was “BVDUC-S”.

###### Diagnosis.

One of the more delicate *Bavia*, along with *B. capistrata*, having legs II–IV very much paler than I, and thus resembling *Indopadilla*. Its distinction from *B. fedor* is slight: the embolus of both appears as a curved and narrowing blade with a series of retrolateral teeth. The teeth are short, triangular, and closely spaced in *B. nessagyna*, but larger in *B. fedor*, appearing as broad pillars whose bases are well separate (photographs of holotype kindly supplied by J. Boone, Bishop Museum). In *B. nessagyna* the teeth are not on the embolus proper but on a TmA that parallels the embolus (Fig. 64). In *B. fedor* it is unclear whether the teeth are on a TmA or on the embolus itself; Prószyński’s illustration (Berry, Beatty and Prószyński 1997) shows no division into two processes, but it may be that they are closely adpressed. Even still, the overall shapes of the embolic division differ: broad at the base in *B. nessagyna* but abruptly narrowing; narrower at the base in *B. fedor* and narrowing more gradually. Epigyne with arcing ridges lateral to the openings in *B. fedor*; without such a ridge in *B. nessagyna*. *B. nessagyna* differs from B. cf. capistrata in the distinct embolus, less contrasting markings, and the abdominal markings more transverse than longitudinal.

**Figures 54–63.**
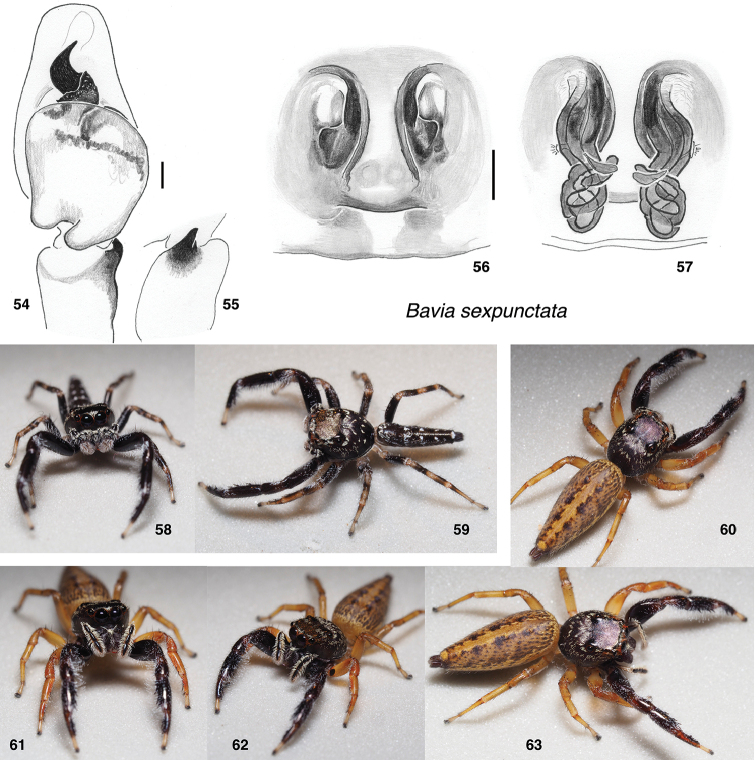
*Bavia sexpunctata*. **54** male left palp, ventral view (Singapore, Upper Peirce Reservoir) **55** same, retrolateral view of tibia **56** epigyne, ventral (Singapore, Bukit Timah) **57** vulva, dorsal **58, 59** male (specimen AS19.2175, Singapore) **60–63** female (specimen AS19.0230, Singapore). Scale bars: on genitalia 0.1 mm.

###### Description.

**Figures 64–75.**
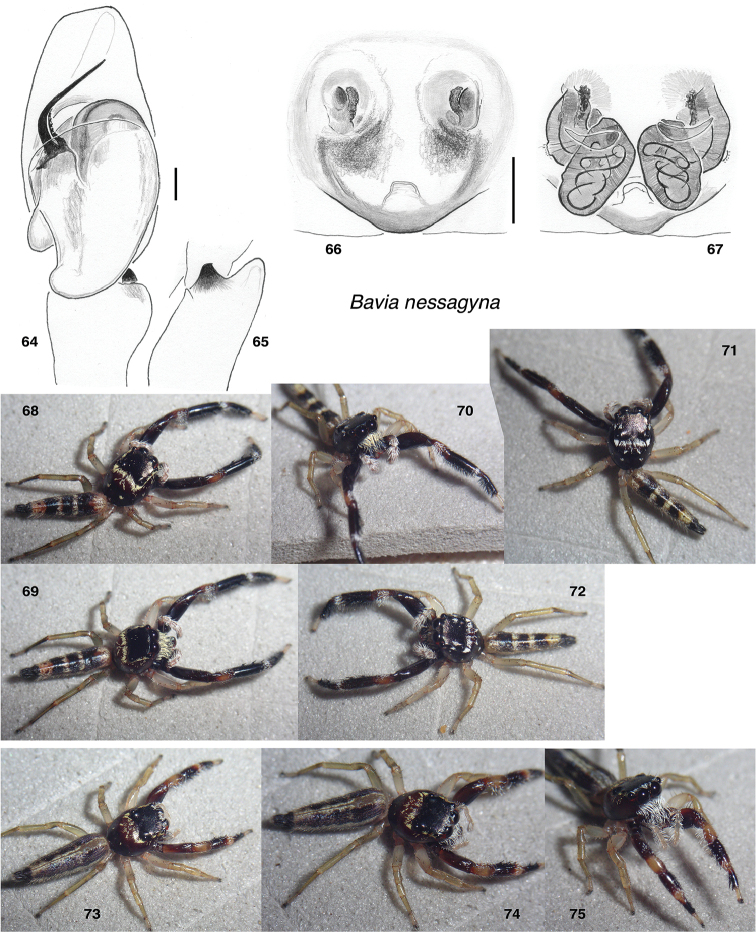
*Bavia nessagyna* sp. nov. **64** male left palp, ventral view (holotype IDWM.20005) **65** same, retrolateral view of tibia **66** epigyne, ventral (specimen IDWM.20003) **67** vulva, dorsal **68, 69** male (specimen SWK12-4726) **70–72** male (specimen SWK12-0590) **73–75** female (specimen SWK12-4087). Distance between substrate grooves 10 mm. Scale bars: on genitalia 0.1 mm.

**Male** (based on holotype, specimen IDWM.20005). Carapace length 3.6; abdomen length 4.8. ***Carapace*** (Fig. 71): Broad, seeming even broader because of small tuft of setae at widest point just posterior to PLEs. Integument of ocular area black, thorax dark brown, except in alcohol pale orange yellow area just behind the ocular area. Bands of cream coloured scales lie just posterior to and lateral to the ocular area; thorax with a few small spots of cream scales, the two posterior ones of which are prominent and well separated, as in *B. capistrata*. ***Clypeus*** dark and glabrous, but a disorderly fringe of long cream setae overhangs the chelicerae (Fig. 70). ***Chelicerae*** simple and mostly vertical, dark, with cream setae that, with those of the clypeus, give the appearance of an unkempt moustache. At least two teeth on retromargin (paratype with 6). ***Palp*** as in Fig. 64; embolus thin and accompanied by toothed TmA. Femur dark basally, but terminally is pale, as are all more distal segments. Endites with thumb-like lobe laterally (Fig. 6). ***Legs*** II–IV notably paler than legs I, which bear annulae of white scales on patella and distally on tibia. Metatarsus I dark; tarsus pale. Ventral fringe of black setae beneath tibia and metatarsus I. ***Abdomen*** with dorsal markings primarily transverse, with four transverse pale bands separated by three dark bands.

**Female** (based on paratype IDWM.20003). Carapace length 3.3; abdomen length 5.9. ***Carapace*** as in male, but lacking tuft at widest point. ***Clypeus*** and ***Chelicerae*** as in male. Five retromarginal teeth. ***Legs*** similar to those of male, but with first leg not quite so dark, and without the ventral fringe so distinctly developed. ***Abdomen*** with only a hint of transverse bands, instead dominated by longitudinal stripes: a narrow central pale band flanked by wide dark bands. ***Epigyne*** with ECP beneath a posteriorly-projecting mound (Figs 66–67).

###### Natural history.

All specimens from Lambir Hills were collected on big-leaved foliage (e.g., palms) except for IDWM.20004 whose collecting record says simply “foliage”.

###### Additional material examined.

One female from Malaysia: Sarawak: Lambir Hills Nat. Pk., Inoue Trail, 4.2002°N, 114.0346°E to 4.2004°N, 114.0342°E, 200 m el. 4 April 2012 Maddison/Piascik/Ang WPM#12-130. One male from Malaysia: Sarawak: Bako Nat. Pk., Mangroves, beach forest, 1.722°N, 110.446°E, 0 m el. 8 March 2012 Maddison/Piascik/Ang/Lee WPM#12-003.

##### Indopadilla

Taxon classificationAnimaliaAraneaeSalticidae

Caleb & Sankaran, 2019

6FD21A6A-8621-59DE-978B-7AE786532BA9

Indopadilla Caleb & Sankaran, 2019. Type species Indopadilla darjeeling Caleb & Sankaran, 2019.

###### Species included.

*Indopadilla annamita* (Simon, 1903), comb. nov., transferred from *Bavia*

*Indopadilla bamilin* Maddison, sp. nov.

*Indopadilla darjeeling* Caleb & Sankaran, 2019

*Indopadilla kahariana* (Prószyński & Deeleman-Reinhold, 2013), comb. nov., transferred from *Bavia*

*Indopadilla kodagura* Maddison, sp. nov.

*Indopadilla insularis* (Malamel, Sankaran & Sebastian, 2015)

*Indopadilla nesinor* Maddison, sp. nov.

*Indopadilla redunca* Maddison, sp. nov.

*Indopadilla redynis* Maddison, sp. nov.

*Indopadilla sabivia* Maddison, sp. nov.

*Indopadilla sonsorol* (Berry, Beatty & Prószyński, 1997), comb. nov., transferred from *Bavia*

*Indopadilla suhartoi* (Prószyński & Deeleman-Reinhold, 2013), comb. nov., transferred from *Bavia*

*Indopadilla thorelli* (Simon, 1901)

*Indopadilla vimedaba* Maddison, sp. nov.

###### Diagnosis.

Front face of chelicerae concave or flat in both males and females, bordered laterally by a sharp ridge (Fig. 5). The ridge projects laterally in some males (e.g., Fig. 98, triangle, or see the male of *I. vimedaba* figured by Prószyński 1987, p. 105, as “*Stagetilus semiostrinus*”). (Males of *Bavia* may have chelicerae with a flat front face, and males of *Padillothorax* may have the front face concave medially, but none have such a sharp or extended lateral ridge.) Clypeus very narrow at centre, exposing a broad expanse of arthrodial membrane that in most species is white (Figs 96, 115, 128; Żabka 1988; Prószyński and Deeleman-Reinhold 2013 fig. 12; Malamel et al. 2015 fig. 9; Caleb et al. 2019 figs. 4, 21; dark in *I. kahariana*, *I. darjeeling*, *I. bamilin*). Spermatheca anterior to copulatory openings (posterior in other baviines). Retromarginal teeth of male not clustered onto a raised hump; rather, the teeth occur on a long sharp ridge that extends medially from base of fang (e.g., Prószyński 1987, p. 105). Male endite rounded, lacking lobe or sharp corner (Fig. 7). Thorax with a bulge just behind the PLE that gives the carapace a “muscular” appearance (Figs 5, 14; see triangle on Fig. 14). (This area contains the muscle attachments for the palps, first legs, and possibly the second legs.) Abdominal markings varied but include a pale longitudinal stripe along each side that is broken approx. half way toward the back, followed by a more posterior pale spot that extends slightly dorsally (Figs 79, 99, 103). When the embolus is long (e.g., Fig. 93), it arises simply as an extension of the tegulum, not being clearly divided from the tegulum as in *Piranthus* and *Maripanthus*.

This distinctive group may have many dozens or even hundreds of species, judging from the rate of discovery of new species among the few specimens being collected. We show in Figs 129–142 some of the apparently-new species that we are not naming. Matching males and females with such sparse collecting can be difficult. We have co-collected both males and females of *I. kahariana*, and they fall together on the molecular phylogeny (Fig. 3). Among the new species, only two are represented by both sexes, *I. redunca* and *I. vimedaba*. We interpret the male *I. bamilin*, *I. kodagura*, and *I. sabivia* as not being matches for the female *I. nesinor* because they are distinct on the molecular phylogeny or have mismatching genitalia (length of embolus vs. copulatory ducts; elaboration of RTA vs. ECP).

Embolus length varies through *Indopadilla*, short in many species, in other species (e.g., *I. kodagura*, *I. suhartoi*) as long as in some *Piranthus* and *Marapathus*. While we might be tempted to split the group into two – *Indopadilla* with a long embolus in South Asia and southeast Asia, and a new genus with a short embolus in southeast Asia – the most complete molecular data nests the long-embolus *I. kodagura* among short embolus species (Fig. 1). Also, even if the long and short embolus species formed mutually monophyletic groups, dividing them would break one very distinctive and easily recognized clade (cheliceral ridge, thoracic bulges, wide arthrodial membrane on face, etc.) into two much more subtle groups. We therefore maintain the whole group as a single distinctive genus.

The peculiar carapace bulge and exposed arthrodial membrane on the clypeus hint to the possibility that *Indopadilla* may use unusual biomechanics. The third leg claw tufts appear noticeably larger than the others. *Indopadilla* are excellent jumpers, difficult to collect even on a beating sheet, from which they can escape in a single decisive bound. They are usually collected from foliage. A video of living males of *I. kodagura* and the undescribed species “BVBTN” (Figs 134–136) is available in Maddison (2020).

##### Indopadilla bamilin

Taxon classificationAnimaliaAraneaeSalticidae

Maddison, sp. nov.

AB5805F3-E60F-5AC2-8AA5-26C669FBCA1D

http://zoobank.org/537ADFA4-923D-4295-8824-D22388ED5BCA

Figs 76–79

###### Type material.

***Holotype*** male (specimen SWK12-1618, in UBCZ) from Malaysia: Sarawak: Mulu Nat. Pk., Clearwater Cave Trail, 4.0597°N, 114.8292°E to 4.0592°N, 114.8291°E, 60 m el. 14 March 2012 Maddison/Piascik/Ang WPM#12-027.

###### Etymology.

An arbitrary combination of letters, ungendered. *Other names*: In Maddison (2015b) and WPM’s lab notebooks the informal code for this species was “BVBML”.

###### Diagnosis.

**Figures 76–79.**
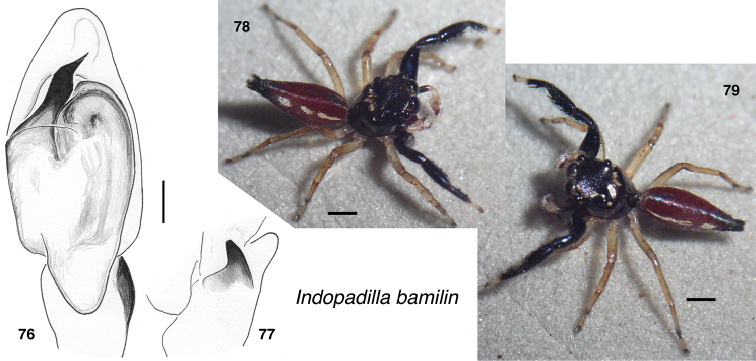
*Indopadilla bamilin* sp. nov., male holotype SWK12-1618 **76** left palp, ventral view **77** same, retrolateral view of tibia **78, 79** holotype. Scale bars: on palp 0.1 mm; on bodies 1.0 mm.

Both embolus and RTA short and simple (Fig. 76). Other species with such a short embolus have a distinct TmA (*I. annamita*, *I. kahariana*, *I. redunca*, *I. sabivia*), which seems lacking in *I. bamilin*. First legs and body dark. None of the known females is an obvious match for this male in terms of markings or molecular phylogenetic placement, but also the unmatched females have longer copulatory ducts than would be expected for such a short embolus, or have a more dramatic ECP than expected for the simple RTA.

###### Description.

**Male** (based on holotype, specimen SWK12-1618). Carapace length 2.3; abdomen length 3.0. ***Carapace*** dark brown, slightly paler around fovea, with a few patches of pale scales on thorax (Figs 78, 79). ***Clypeus*** dark, narrow; status of arthrodial membrane unclear, as chelicerae are somewhat sunken into the prosoma. ***Chelicerae*** vertical, brown, concave in front and with lateral ridge. Retromarginal teeth at least three, on long ridge. ***Palp*** femur dark; more distal segments pale. Embolus short, undivided (i.e., no TmA; Fig. 76). Tibia with dorsal lobe distally (Fig. 77). Endite rounded laterally, without corner or lobe. ***Legs*** strongly contrasting between black leg I and pale legs II–IV. First patella entirely black. ***Abdomen*** dark red in life, with longitudinal band of pale scales on each side broken as typical for the genus.

##### Indopadilla kahariana

Taxon classificationAnimaliaAraneaeSalticidae

(Prószyński & Deeleman-Reinhold, 2013) comb. nov.

A1A082C7-0F2A-5A81-9A37-927F32EDEF9A

Figs 22 , 30 , 80–92

Bavia kahariana Prószyński & Deeleman-Reinhold, 2013: 115–117, figs 1–9.

###### Notes.

We include illustrations of the described *Indopadilla kahariana*, to show living specimens and to show the genitalia in detail. Christa Deeleman-Reinhold kindly compared the type specimens to our illustrations and confirmed the match. A photograph of a living female is shown by Koh and Bay (2019, page 203, fig. A). In WPM’s field and lab notebooks the informal code for this species was “BVSMB”.

##### Indopadilla kodagura

Taxon classificationAnimaliaAraneaeSalticidae

Maddison, sp. nov.

36D9E5A3-CDAF-5129-A7C2-A0F89264FB8B

http://zoobank.org/013153B4-32EC-4F6A-A921-E7BB652AF67A

Figs 93–99

###### Type material.

***Holotype*** male (NCBS-BN351, also known as AS19.4314), in NCBS collection, from India: Karnataka: Kodagu: Yavakapadi, Honey Valley area, forest & edge, 12.215°N, 75.659°E to 12.216°N, 75.661°E, 1300 m elev. 25 June 2019 W. Maddison & K. Marathe WPM#19-077.

###### Etymology.

In the Kodava language, *kodagura* means from Kodagu. *Other names*: In WPM’s field or lab notebooks the informal code for this species was “BVHVW”.

###### Diagnosis.

Very similar to *I. insularis*, contrastingly marked in dark brown and yellow, with the face appearing white because the clypeus is withdrawn toward the eyes to expose a bright white arthrodial membrane. Like *I. insularis*, *I. darjeeling*, *I. sonsorol*, *I. suhartoi*, and *I. thorelli* in having a long thin embolus, but even longer than in those species, arising from the retrolateral basal corner of the bulb.

###### Description.

**Male** (based on holotype, specimen NCBS-BN351). Carapace length 2.6; abdomen length 4.0. ***Carapace*** integument dark brown to black except to either side of fovea and narrow medial stripe on thorax, and with small patches of yellow scales in pattern typical for *Indopadilla*. ***Clypeus*** dark, extremely narrow, exposing white arthrodial membrane. ***Chelicerae*** dark, with lateral ridge bearing a tooth (Fig. 98, triangle). At least five retromarginal teeth, on long ridge. ***Palp*** yellow except for black cymbium. Embolus simple, long (Fig. 93); RTA a flat blade. ***Legs*** strongly contrasting between dark brown to black first legs, and yellowish posterior legs. Darkness of first legs relieved by pale tarsus, and honey coloured path on patella. ***Abdomen*** black except for two prominent yellow-white patches along each side.

###### Natural history.

A video of the living holotype is available in Maddison (2020).

**Figures 80–92.**
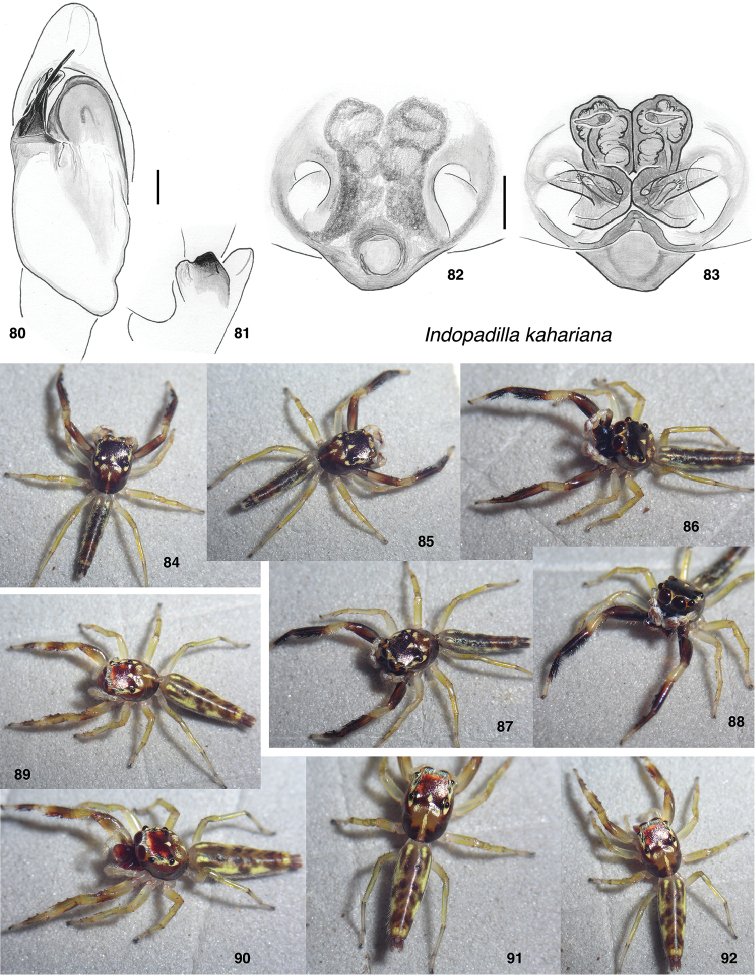
*Indopadilla kahariana*. **80** male left palp, ventral view (Mulu Nat. Pk., 4.045°N, 114.816°E) **81** Same, retrolateral view of tibia **82** epigyne, ventral (specimen SWK12-1876) **83** vulva, dorsal **83–88** male (SWK12-1163) **89–92** Female (SWK12-1876). Distance between substrate grooves 10 mm. Scale bars: on genitalia 0.1 mm.

**Figures 93–99.**
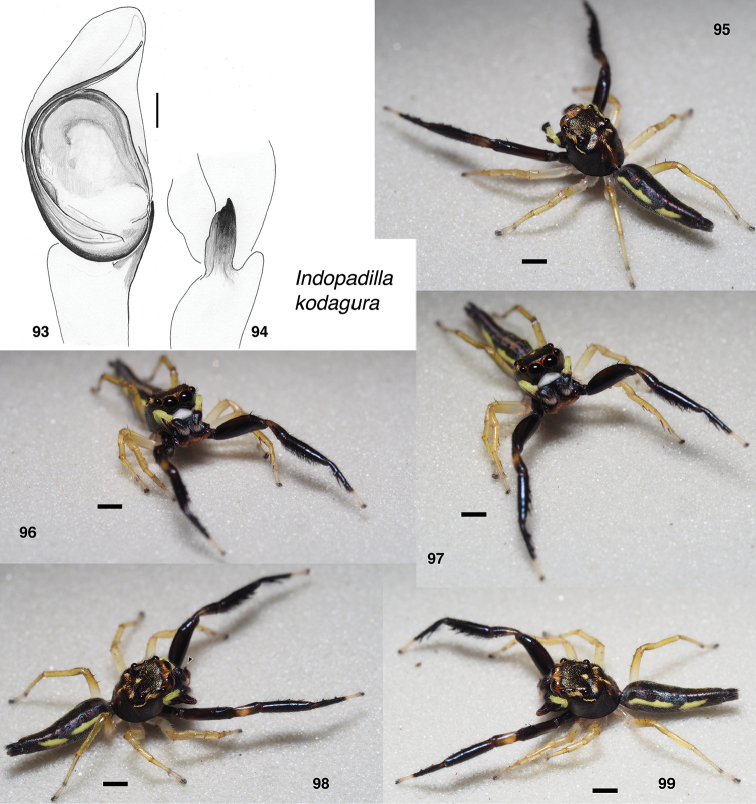
*Indopadilla kodagura* sp. nov., holotype. **93** male left palp, ventral view **94** same, retrolateral view of tibia **95–99** male. Scale bars: on palp 0.1 mm; on bodies 1.0 mm.

##### Indopadilla nesinor

Taxon classificationAnimaliaAraneaeSalticidae

Maddison, sp. nov.

A2865BB8-C1A1-5D45-8741-964259B4E4A4

http://zoobank.org/C34E99A9-07C6-4ECD-976A-3FEA32CB0E98

Figs 100–103

###### Type material.

***Holotype*** female (specimen MRB076), in LKCNHM, from Singapore: Nee Soon Swamp Forest, beating vegetation, 1.39°N, 103.81°E, 12 May 2005. W. Maddison, D. Li, I. Agnarsson, J. X. Zhang. WPM#05-015. ***Paratype*** female (specimen JK.14.05.19.0015) from Singapore: Central Catchment Nature Reserve Upper Peirce Reservoir 1.3811°N, 103.8156°E, J. K. H. Koh 19 May 2014.

**Figures 100–103.**
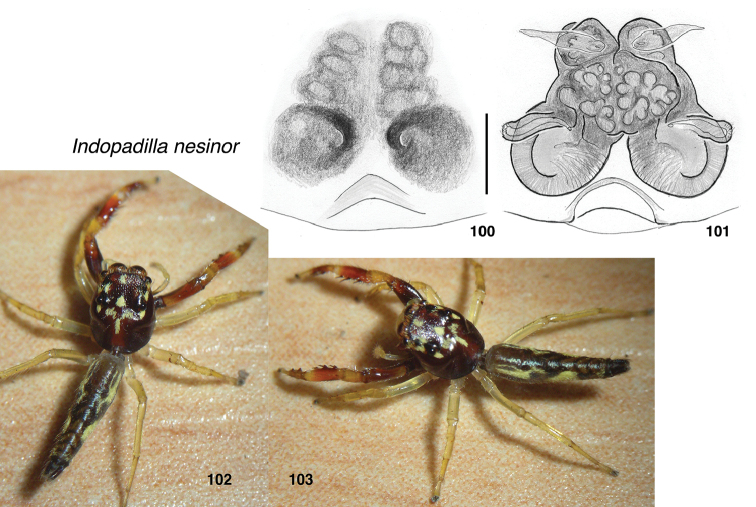
*Indopadilla nesinor* sp. nov., female holotype MRB076 **100** epigyne, ventral **101** vulva, dorsal **102, 103** Body. Scale bars: on epigyne 0.1 mm.

###### Etymology.

An arbitrary combination of letters, ungendered. *Other names*: In WPM’s lab notebooks the informal code for this species was “BVNES”.

**Figures 104–112.**
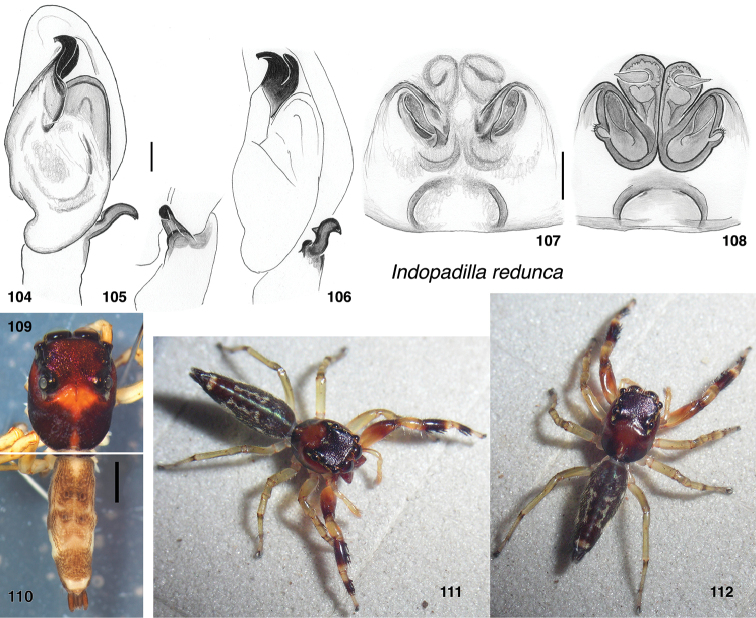
*Indopadilla redunca* sp. nov. **104** male left palp, ventral view (holotype SWK12-M0009) **105** same, retrolateral view of tibia **106** same, oblique ventral-retrolateral view **107** epigyne, ventral (specimen SWK12-1831) **108** Vulva, dorsal **109** male holotype, carapace **110** same, abdomen **111, 112** Female (SWK12-1831). Distance between substrate grooves 10 mm. Scale bars: on genitalia 0.1 mm.

###### Diagnosis.

As in *I. vimedaba*, the face appears white because of the exposed arthrodial membrane, whose boundary with the very narrow clypeus is indistinct. Differs from *I. vimedaba* in having chevroned abdominal markings visible in alcohol and epigynal openings small and copulatory ducts densely tangled and fused (Fig. 101).

###### Description.

**Female** (based on holotype, specimen MRB076). Carapace length 2.5; abdomen length 3.6. ***Carapace*** integument in alcohol dark brown, paler in ocular area and along midline of thorax, with patches of yellow scales (Fig. 102). ***Clypeus*** extremely narrow, but broad arthrodial membrane is exposed and bright white. ***Chelicerae*** brown, with concave front and lateral ridge. At least four retromarginal teeth. ***Legs*** pale except first, whose femur is brown darkening to black ventrally, and whose tibia and metatarsus are red-brown in the middle. ***Abdomen*** dark except for large patches of yellow scales along each side. ***Epigyne*** with shallow ECP and small openings (Fig. 100).

##### Indopadilla redunca

Taxon classificationAnimaliaAraneaeSalticidae

Maddison, sp. nov.

8E6AD145-3FAE-517F-84A7-92047DE371AE

http://zoobank.org/F3D55C28-3A7A-48CC-82FA-D91D0A363106

Figs 5 , 7 , 14 , 104–112

###### Type material.

All from Malaysia: Sarawak: Mulu Nat. Pk. ***Holotype.*** Female (specimen IDWM.20011, in UBCZ), from Botanical Trail, 4.0380°N, 114.8137°E, 50 m el. 18 March 2012 W. Maddison WPM#12-059. ***Paratype*** female (specimen SWK12-1831, in UBCZ) from Botanical Trail, 4.0404°N, 114.8151°E to 4.0405°N, 114.8154°E, 50 m el. 16 March 2012 Maddison/Piascik/Ang WPM#12-039. ***Paratype*.** Male (specimen SWK12-M0009, in UBCZ) from Nightwalk Trail, 4.0446°N, 114.8154°E to 4.0450°N, 114.8156°E, 50 m el. 24 March 2012 Maddison/Piascik/Ang WPM#12-090. ***Paratype*** male (specimen JK.12.01.22.0024, in LKCNHM) from Sungai Paku Waterfall Trail, 04.0372°N, 114.8247°E, J. K. H. Koh 22 January 2012.

###### Etymology.

Latin, meaning bent backward, referring to both the RTA and the ridge in front of the epigynal openings. *Other names*: In Maddison (2015b) and WPM’s field or lab notebooks the informal code for this species was “BVMTT”. In J. Koh’s notebooks it was referred to as “Bent RTA”.

###### Notes.

The living paratype male 12.01.22.0024 is shown as figure E on p. 203 of Koh and Bay 2019.

###### Diagnosis.

Male palp distinctive for bent RTA and thick curled embolus accompanied by TmA (Fig. 104). Epigyne similar to that of *I. redynis*, with cavernous ECP, but with each opening framed by an anterior curved ridge. Posterior third of abdomen dark except for prominent pale transverse band just in front of anal tubercle. First femur pale basally, lacking dark spot of *I. redynis*.

###### Description.

**Male** (based on paratype, specimen SWK12-M0009). Carapace length 2.9; abdomen length 3.6. ***Carapace*** in alcohol dark brown except medium brown near fovea, and a small medium brown stripe medially on thoracic slope. ***Clypeus*** dark, glabrous, very narrow in middle, exposing a white arthrodial membrane beneath. ***Chelicerae*** dark, concave and with lateral ridge. At least four retromarginal teeth, on long sharp ridge. ***Palp*** pale except dark gray retrolateral face of femur. Embolus thick and curved, accompanied by similarly curved TmA (Figs 104, 106). RTA projects out away from axis of palp, curved toward the proximal (Figs 105 106). ***Legs*** strongly contrasting between the first legs (dark, except for patella, tarsus, and much of femur). ***Abdomen*** as described under Male-female matching.

**Female** (based on holotype, specimen IDWM.20011). Carapace length 3.2; abdomen length 4.1. ***Carapace*** dark brown, paler around fovea and in a narrow medial band on thoracic slope. ***Clypeus*** narrow, dark, glabrous, exposing white arthrodial membrane. ***Chelicerae*** vertical, concave in front. Five retromarginal teeth. ***Legs*** pale except for first leg, whose tibia and metatarsus are dark brown in the middle, and the femur which grades to dark brown terminally. ***Abdomen*** marked as described under Male-female matching. ***Epigyne*** (Figs 107, 108) with large ECP medially. Openings behind arcing ridges.

###### Male-female matching.

The male and female, both collected at Mulu National Park, are matched primarily on the basis of markings and expected genitalic correlations. They share abdominal markings (Figs 110–112): just in front of the anal tubercle is a prominent pale transverse mark, more prominent than in other species, and in front of the pale mark is a dark more or less unmarked area that extends to cover the posterior third of the abdomen. Just in front of that, in the middle third, are two uneven narrow broken longitudinal bands, besides which are similar uneven pale bands on the side. While other *Indopadilla* species have uneven longitudinal bands, no others known have the bands so restricted by a dark posterior third, nor have the pale pre-anal band so prominent. In genitalia, the large ECP is expected to correspond to a dramatic RTA, which the matched male has. A female was chosen as holotype as it is in best condition, and the molecular data are from a matching female.

##### Indopadilla redynis

Taxon classificationAnimaliaAraneaeSalticidae

Maddison, sp. nov.

260DDD79-844C-5E9A-B831-9F2AD15A6DFD

http://zoobank.org/66E4CC75-6E34-479F-9A02-D304027C9EB5

Figs 113–116

###### Type material.

***Holotype*** female (specimen IDWM.20012, in UBCZ) from Malaysia: Sarawak: Kubah Nat. Pk., roadside, 1.603–4°N, 110.185°E, 350 m el. 7 March 2012 Maddison/Piascik/Ang/Lee WPM#12-002. ***Paratype*** female (specimen SWK12-0080, in UBCZ) from Malaysia: Sarawak: Kubah Nat. Pk., Waterfall Trail, 1.605–6°N, 110.185–7°E, 300 m el. 7 March 2012 Maddison/Piascik/Ang/Lee WPM#12-001.

**Figures 113–116.**
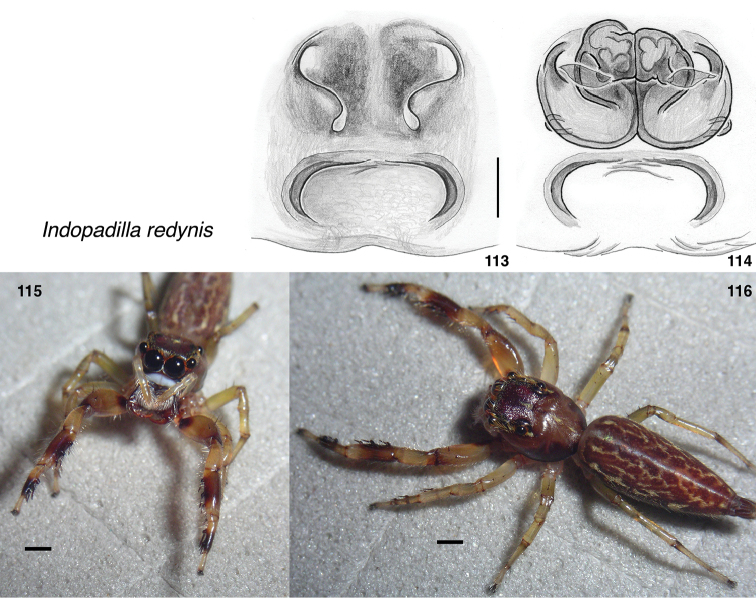
*Indopadilla redynis* sp. nov. **113** epigyne, ventral (holotype IDWM.20012) **114** vulva, dorsal **115, 116** Female (SWK12-0080). Scale bars on epigyne 0.1 mm; on bodies 1.0 mm.

###### Etymology.

An arbitrary combination of letters, ungendered. *Other names*: In WPM’s lab notebooks the informal code for this species was “BVMT2”; it was also sometimes confused with *I. redunca* and labelled “BVMTT”.

###### Diagnosis.

Similar in colour and epigyne to *I. redunca*. The epigyne differs in having the anterior part of the cavernous ECP with a more sharply defined boundary, the openings not behind a curved ridge, and the edge of the opening clearly sinuate (Fig. 113). The abdominal dorsum has fine reticulate markings throughout, not showing the unmarked posterior third. First leg markings match those of *I. redunca* except that anterior base of first femur with dark patch. Pale area on thoracic slope much broader than in *I. redunca*.

###### Description.

**Female** (based on holotype, specimen IDWM.20012). Carapace length 3.6; abdomen length 5.7. ***Carapace*** integument medium to dark red-brown, except upper part of thorax between and beside eyes, continuing as a broad medial pale area to pedicel. ***Clypeus*** dark, extremely narrow, exposing broad white arthrodial membrane. ***Chelicerae*** light brown, concave in front. Five teeth on retromargin. ***Legs*** pale except dark brown on middle of first tibia and metatarsus, and darker patch at front base of first femur. ***Abdomen*** red-brown, with delicate reticulate pale markings. ***Epigyne*** (Fig. 113) with large cavernous ECP; openings sinuate, resembling those of dendryphantines.

##### Indopadilla sabivia

Taxon classificationAnimaliaAraneaeSalticidae

Maddison, sp. nov.

F30416C9-AF55-5699-9740-408DA2B8E6F6

http://zoobank.org/28AEEB1A-86D2-4F38-B400-D4C1C4D089DC

Figs 117–120

###### Type material.

***Holotype*** male (specimen d107, in UBCZ) from Malaysia: Sabah: Village of Kiabau. Central Sabah. 5.8315°N, 117.2245°E, 23 November 2000 K. Ober #00.437.

###### Etymology.

An arbitrary combination of letters, ungendered. *Other names*: In WPM’s lab notebooks the informal code for this species was “BVSAB”.

###### Diagnosis.

Palp similar to *I. kahariana* with a short sharp embolus with a TmA behind it, but the TmA is broader and more retrolaterally placed than in *I. kahariana*. RTA with sharp point, unlike the broad flat RTA of *I. kahariana*. These two species are sister groups on the molecular phylogeny (Fig. 1).

###### Description.

**Figures 117–120.**
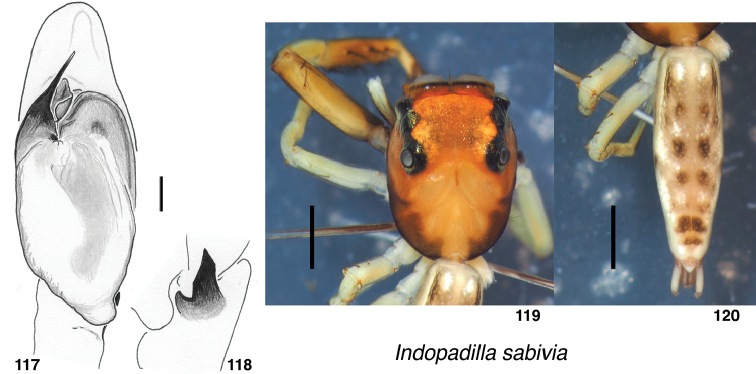
*Indopadilla sabivia* sp. nov., male holotype (specimen d107) **117** left palp, ventral view **118** same, retrolateral view of tibia **119, 120** Body. Scale bars: on palp 0.1 mm; on body 1.0 mm.

**Male** (based on holotype, specimen d107). Carapace length 2.8; abdomen length 3.8. Overall appearance similar to that of *I. kahariana*, paler coloured than many male *Indopadilla*, honey to medium brown. ***Carapace*** medium brown to honey coloured, darker on sides, with ocular area and larger portion of dorsal thorax quite pale. ***Clypeus*** narrow, exposing white arthrodial membrane. ***Chelicerae*** brown, with lateral ridge. Six retromarginal teeth, on long ridge. ***Palp*** pale except basal part of femur. Embolus short and sharp, with broad TmA behind (Fig. 117). ***Legs*** mostly honey coloured, first somewhat darker and with ventral fringe on patella through metatarsus. ***Abdomen*** in alcohol similar to that of female *I. kahariana*, with series of darker paired spots dorsally and elongated pale spots along the side.

##### Indopadilla vimedaba

Taxon classificationAnimaliaAraneaeSalticidae

Maddison, sp. nov.

422FE007-D7F0-5C68-B193-022BA07223B9

http://zoobank.org/28AEEB1A-86D2-4F38-B400-D4C1C4D089DC

Figs 121–128

Stagetilus semiostrinus : Prószyński, 1987: figs on pages 105–105 (misidentification).
Padillothorax semiostrinus : Prószyński 2018: 174–175, figs 28F–L (misidentification).

###### Type material.

***Holotype*** male (specimen JK 13.09.03.0011), in LKCNHM, from Singapore: Nee Soon Swamp, 2 September 2013. J. K. H. Koh.

###### Etymology.

An arbitrary combination of letters, ungendered. *Other names*: In Maddison (2015b) and WPM’s field or lab notebooks the informal code for this species was “BVMDB”.

###### Notes.

As noted under *Padillothorax*, this species was mistaken for *Padillothorax semiostrinus*, and illustrated by Prószyński (1987) under that name. He illustrates a male and female in the same vial 15151 in the MNHN Paris from the “Malayana” peninsula.

###### Diagnosis.

**Figures 121–128.**
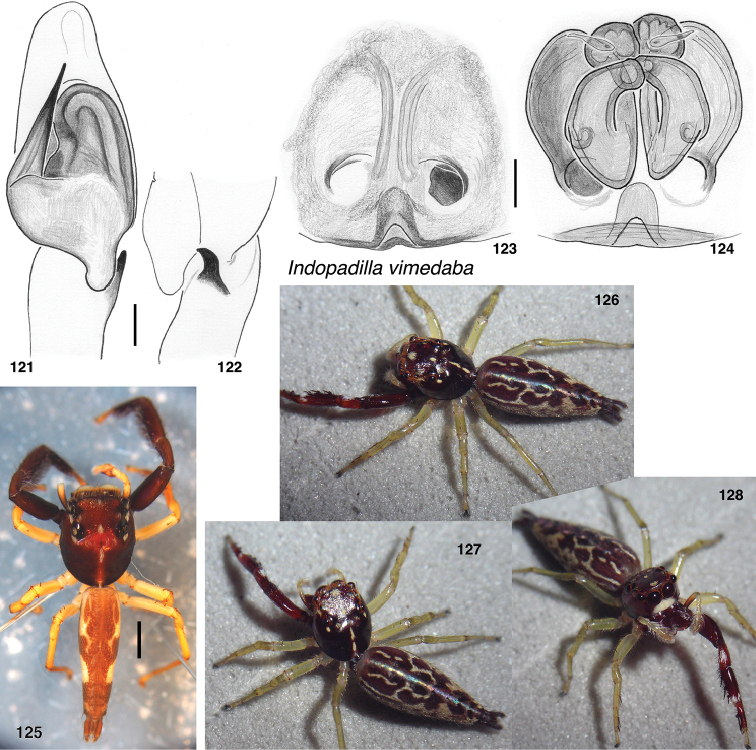
*Indopadilla vimedaba* sp. nov. **121** male left palp, ventral view (holotype JK.13.09.03.0011) **122** same, retrolateral view of tibia **123** epigyne, ventral (specimen SWK12-3620) **124** vulva, dorsal **125** holotype male **126–128** female SWK12-3620. Scale bars: on genitalia 0.1 mm; on bodies 1.0 mm.

Palp with embolus longer that half the length of the bulb, straight and tapering (Fig. 121). Other *Indopadilla* have the embolus either longer and curved, or shorter. Epigyne with small but distinct ECP on back margin medially; openings simple and posterior (Fig. 123). Body dark brown except for highly contrasting but narrow pale cream markings, reticulate on abdomen.

###### Description.

**Male** (based on holotype, specimen JK 13.09.03.0011). Carapace length 2.4; abdomen length 3.5. ***Carapace*** in alcohol dark brown to black, slightly paler around fovea and in narrow medial band along thoracic slope, which also has some white scales; other patches of white scales along borders of ocular area. ***Clypeus*** extremely narrow at centre, and beneath it is a broad expanse of white arthrodial membrane; precise boundary between the clypeus and arthrodial membrane indistinct. ***Chelicerae*** with strong lateral ridge that extends into a flange near the fang, as drawn by Prószyński (1987: 105). Three promarginal teeth and at least five retromarginal. ***Palp*** honey coloured except darker base of femur. Embolus a narrow long triangle (Fig. 121). ***Legs*** honey coloured except much larger first leg, which bears a ventral fringe. ***Abdomen*** dark above and below, with two pair of lateral pale spots, the anterior of which connects with a few thin pale lines.

**Female** (based on specimen SWK12-3620). Carapace length 3.3; abdomen length 5.4. ***Carapace*** integument in alcohol medium red-brown except for narrow medial pale band along thoracic slope. ***Clypeus*** extremely narrow, exposing broad white arthrodial membrane (Fig. 128). ***Chelicerae*** concave in front with lateral ridge. At least four retromarginal teeth. ***Legs*** pale except first leg, dark except tarsus. ***Abdomen*** brown with distinct reticulation of pale scales. ***Epigyne*** (Fig. 123) with simple openings, from which broad copulatory ducts proceed anteriorly before narrowing considerably and proceeding to the posterior, then back anteriorly to the spermathecae.

###### Male-female matching.

The male and female described above were not co-collected, but they match well the male and female (MNHN 15151, photographs examined) described by Prószyński, which were in the same vial, and thus likely co-collected. These males and females match in markings: the first leg is all dark except the tarsus; the abdominal dorsum is dark with some narrow pale lines, the venter dark; the pale medial band of the integument of the thoracic slope is narrow; there is a narrow band of pale scales on the midline low on the thoracic slope; the triangle of scales near the fovea is narrow. The male has fewer lines in its abdominal markings than the female, but those it has are precise matches to the female. Other *Indopadilla* differ; e.g., another candidate female, *I. nesinor*, has the first leg considerably more strongly banded. suggesting her male should have a first patella paler than seen in Fig. 125, more like that of *I redunca* (Koh and Bay 2019:. 203, fig. E), whose female’s leg is banded similarly to *I. nesinor*. Also, *I. nesinor* differs in having a pale underside of the abdomen, and different thoracic markings. The relatively long but wide embolus of the male is a good fit to the openings and first broad part of the duct of the matched female, being ~ 0.07 mm wide at its base and ~ 0.3 mm long. In contrast, the openings of *I. nesinor* are only ~ 0.02 mm wide, enough to accommodate only a small portion of the embolus.

**Figures 129–142.**
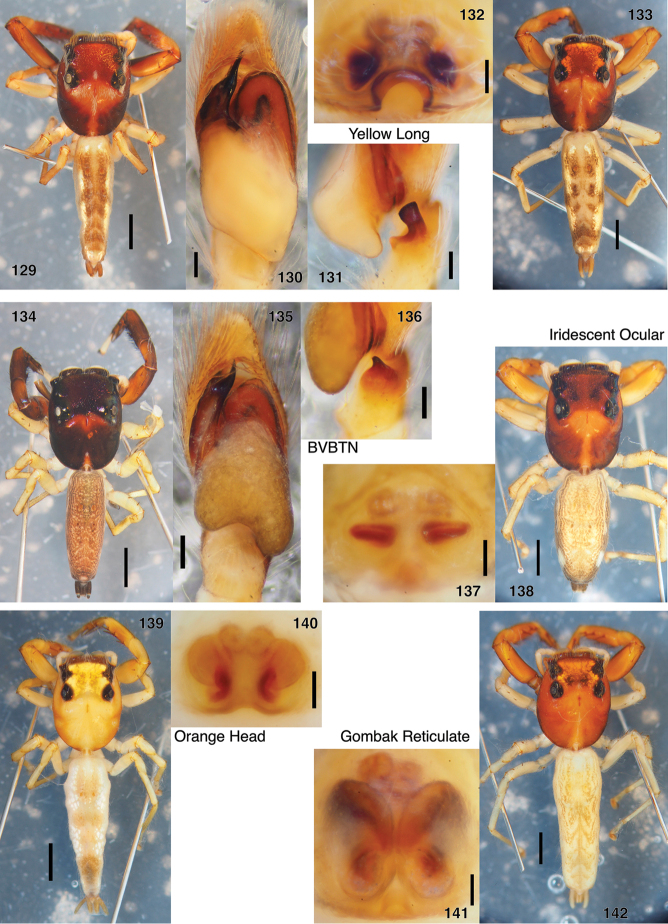
Unidentified or undescribed *Indopadilla*, all in LKCNHM except **134–136**, in UBCZ. **129–131** male “Yellow Long” (specimen JK08.04.29.0029, Brunei, 4.6044°N, 114.6450°E), body, ventral palp, retrolateral palp tibia **132, 133** female “Yellow Long” (specimen JK.12.03.14.0031, Brunei, 4.7036°N, 114.6264°E), epigyne, body **134–136** male “BVBTN” (specimen AS19.2286, Singapore, 1.3562°N, 103.7748°E to 1.3572°N, 103.7734°E), body, ventral palp, retrolateral palp tibia **137, 138** female “Iridescent Ocular” (specimen JK.13.02.16.3005, Brunei 4.5764°N, 115.0731°E), epigyne, body **139, 140** female “Orange Head” (specimen JK.12.03.21.0011, Brunei, 4.5833°N, 114.5047°E), body, epigyne (shown alive as fig. B on p. 203 of Koh and Bay 2019) **141, 142** female “Gombak Reticulate” (specimen JK.98.03.05.0001, Singapore, 1.3619°N, 103.7592°E), epigyne, body. Scale bars: on genitalia 0.1 mm; on bodies 1.0 mm.

###### Additional material examined.

One female (specimen SWK12-3620, in UBCZ) from Malaysia: Sarawak: Mulu Nat. Pk., headquarters area, on & in buildings, 4.042°N, 114.814°E, 50 m el. 26–27 March 2012 Maddison/Ang WPM#12-100.

##### Stagetillus

Taxon classificationAnimaliaAraneaeSalticidae

Simon, 1885

E21D83C2-CCB6-588E-8E42-9424ACF34FD6

Stagetillus Simon, 1885. Type species S. opaciceps Simon, 1885.
Hyctiota Strand, 1911, syn. nov. Type species H. banda Strand, 1911.

###### Species included.

*Stagetillus banda* (Strand, 1911), comb. nov.

*Stagetillus opaciceps* Simon, 1885

*Stagetillus irri* Maddison, sp. nov.

###### Notes.

*Hyctiota banda* Strand, 1911 is based on a juvenile (SMF 2511 in SMF, examined) and not easily placed. Its being a baviine is suggested by its elongate body (most like that of *Indopadilla* or *Stagetillus*), plurident chelicerae, and Asian origin. Its macrosetae sockets resemble those of baviines (Figs 21–23). It lacks the thoracic bulge and ridged chelicerae of *Indopadilla*, and the flat carapace of *Padillothorax*. Like *Stagetillus*, the fovea is displaced slightly to the posterior. The two dark bands running through the PLE shown in Strand’s (1911) original figure (no longer visible in specimen) resemble those of male *S. opaciceps*. The carapace is widest posteriorly, though not so clearly as in Figs 15 and 16. Although its synonymy with *Stagetillus* is not certain, by placing it with *Stagetillus*, a salticid genus otherwise incertae sedis is provisionally settled.

###### Diagnosis.

Carapace distinctive in shape, widest point toward the posterior, approx. half way between the back eyes and the pedicel, and in colour, orange or yellow with the white digestive diverticulum showing beneath the transparent ocular quadrangle. Palp much like that of *Bavia*, with short blade-like embolus.

##### Stagetillus opaciceps

Taxon classificationAnimaliaAraneaeSalticidae

Simon, 1885

AF6389BD-82C6-5C13-88F6-91AFD766E090

Figs 8 , 15 , 16 , 23 , 31 , 143–150

Stagetillus opaciceps Simon, 1885: 32.

###### Note.

**Figures 143–150.**
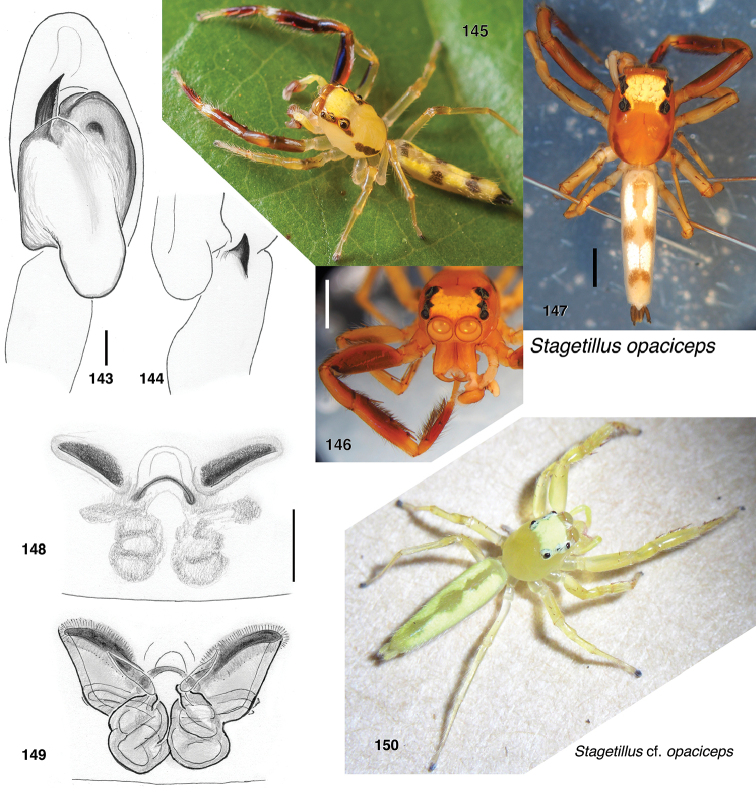
*Stagetillus opaciceps* male, and a female tentatively identified as *S. opaciceps*. **143** Male left palp, ventral view (specimen JK.13.02.26.0017) **144** same, retrolateral view of tibia **145** living male from Belait, Brunei (photograph Joseph Koh 2019) **146, 147** male (specimen JK.08.08.19.0001) **148** female epigyne (specimen MRB079), ventral **149** vulva, dorsal **150** living female. Scale bars: on genitalia 0.1 mm; on bodies 1.0 mm.

Prószyński’s (1987) illustrations of Simon’s type specimen characterize the male well. Two additional males illustrated here (Figs 143–147) are conspecific or at least very close (the male from Brunei, Figs 146, 147, has a slightly narrower embolus). Females of the species have not been reported, although we now tentatively identify, as the female of *S. opaciceps*, the specimen MRB079 from Ulu Gombak used by Maddison et al. (2014) for molecular phylogeny (Figs 148–150). That the female is a *Stagetillus* is suggested by its similarities to the male in size and body form, carapace shape, yellow coloration, and visibility of the ocular digestive diverticulum. The modest ECP and epigynal openings are what might be expected from the small simple RTA and embolus of the known male of *S. opaciceps*. Given that Ulu Gombak is only 70 km from a known male of *S. opaciceps* (Figs 143, 144), the match of the Ulu Gombak female to *S. opaciceps* is credible. We therefore label the female, used here for molecular data, as S. cf. opaciceps (Figs 148–150). In WPM’s lab notebooks the informal code for the female was “STULG”.

###### Material examined.

Male (specimen JK.13.02.26.0017, in LKCNHM) Malaysia: Negeri Sembilan, Hutan Lipur Ulu Bendul, 2.73°N, 102.0789°E, J. K. H. Koh 26 February 2013; Male (JK.08.08.19.0001, in LKCNHM) Brunei: Belait, Disturbed forest off Labi Road, 4.5858°N, 114.5067°E, J. K. H. Koh 19 August 2008. Female (specimen MRB079, in UBCZ) Malaysia: Selangor: Ulu Gombak Field Station. 3.325°N, 101.753°E, 250 m el. 16–19 May 2005. W. Maddison, D. Li, I. Agnarsson, J. X. Zhang, WPM#05-026.

##### Stagetillus irri

Taxon classificationAnimaliaAraneaeSalticidae

Maddison, sp. nov.

6B14FEE5-A08F-5E1F-A9F4-1C2FB55A4068

http://zoobank.org/012F0D17-8A6A-4CE0-8EEB-8F0A3D2562AA

Figs 151–157

###### Type material.

***Holotype*** male (specimen IDWM.20023, in FSCA) and ***paratype*** female from Philippines: Luzon: Laguna Province, Los Baños, International Rice Research Institute, February 1993. R.R. Jackson (batch RRJ Ph 246/93). Other ***paratypes*** from same locality are 1 male (specimen IDWM.20022), December 1996, R.R. Jackson (batch RRJ Ph 292/96, in UBCZ); 3 females (specimens IDWM.20014, S202, and S203, in UBCZ), 1–16 December 1993, R.R. Jackson & G.B. Edwards; and 2 males 3 females, March 1993, R.R. Jackson (batch RRJ Ph 323/93, in FSCA).

###### Etymology.

From the acronym for the type locality. *Other names*: This species was referred to by Maddison and Hedin 2003 as “unident. (Phil.)” In WPM’s lab notebooks the informal code for this species was “STPHL”.

###### Notes.

The FSCA holds more material of this species, including from Mt. Makiling, near the type locality but at higher elevation (G. B. Edwards, pers. comm.).

###### Diagnosis.

Similar in overall appearance to *S. opaciceps*, from which it differs in palp (narrower and longer embolus) and epigyne (shorter openings and having a central mound, presumably bearing the ECP). Male lacking the dense fringe beneath the first leg (Figs 155 vs. 146), but with a spur on the paturon (Fig. 155, arrow) that is lacking in *S. opaciceps* (Fig. 146). Male endite lacks the lateral bulge seen in *S. opaciceps*.

###### Description.

**Figures 151–157.**
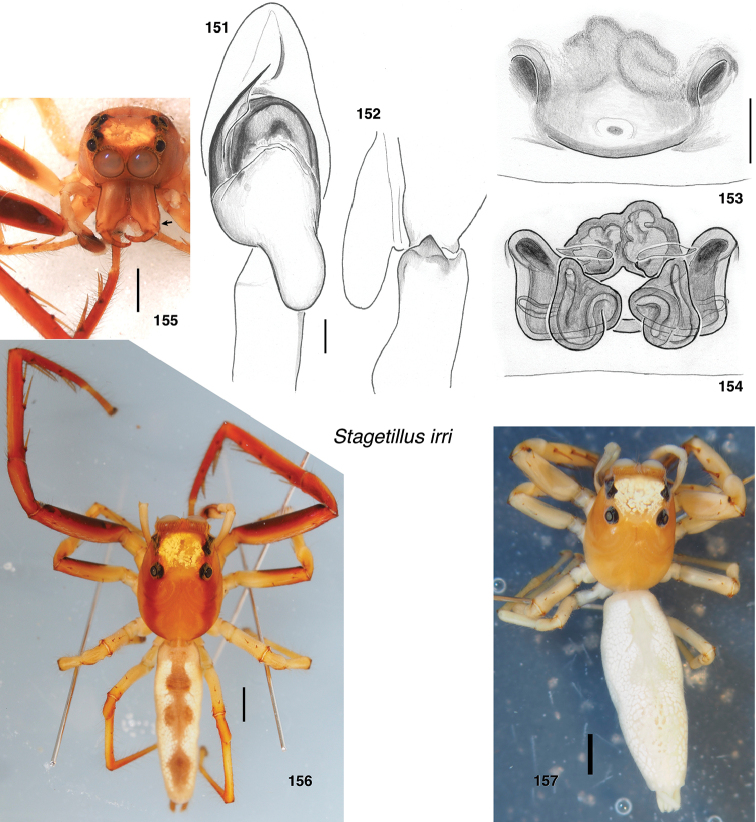
*Stagetillus irri* sp. nov. **151** male paratype IDWM.20022, left palp, ventral view **152** same, retrolateral view **153** female paratype S202, epigyne, ventral **154** same, vulva, dorsal. **155–156** male holotype IDWM.20023 **157** female paratype IDWM.20014. Scale bars: on epigyne 0.1 mm; on body 1.0 mm.

**Male** (based on holotype, specimen IDWM.20023). Carapace length 3.3; abdomen length 4.6. ***Carapace*** yellow-orange with two darker stripes passing along PME, PLE, and to posterior margin, with transparent ocular areas showing bright white digestive diverticular beneath. Shape as in *S. opaciceps* (Fig. 150). ***Clypeus*** narrow, more or less glabrous. ***Chelicerae*** orange, projecting slightly, with a spur on each paturon just above the fang base (arrow, Fig. 155). ***Palp*** pale except darker cymbium. Embolus arising at ~ 9:00 on the bulb, narrowing to a fine tip, with transparent flange retrolaterally (Fig. 151). Long lobe of tegulum overhangs the tibia. RTA broad and obtuse. ***Legs*** pale yellow to dark orange, first pair darkest, with femora of first two pair darker below. First leg long, with only a weak fringe beneath. ***Abdomen*** with dark central band with three wider areas, flanked by bright white.

**Female** (based on paratype, specimen IDWM.20014). Carapace length 2.9; abdomen length 5.2. Entirely light in colour, from white to medium orange, except for the black of eyes, and small black patch at the front distal tip of the first leg femur. ***Carapace*** pale orange, with transparent ocular area showing bright white digestive diverticular beneath. Shape as in *S. opaciceps* (Fig. 157). ***Chelicerae*** orange, with five retromarginal teeth. ***Legs*** pale to dark honey-orange, except for black on prolateral tip of femur I. ***Abdominal*** integument transparent in alcohol, showing white digestive glands to either side of beige heart and mottled stercoral pocket. ***Epigyne*** (Fig. 153) similar to those of *Bavia*, with sclerotized openings on either side of a central mound.

#### Genus *Padillothorax*

##### Padillothorax

Taxon classificationAnimaliaAraneaeSalticidae

Simon, 1901

E0B41E3F-FF99-58C5-9374-453388C74419

Padillothorax Simon, 1901. Type species Padillothorax semiostrinus Simon, 1901.
Bavirecta Kanesharatnam & Benjamin, 2018, syn. nov. Type species Bavirecta flavopuncta Kanesharatnam & Benjamin, 2018.

###### Species included.

*Padillothorax badut* Maddison, sp. nov.

*Padillothorax casteti* (Simon, 1900), comb. nov., transferred from *Bavirecta*

*Padillothorax exilis* (Cao & Li, 2016), comb. nov., transferred from *Bavirecta*

*Padillothorax flavopunctus* (Kanesharatnam & Benjamin, 2018), comb. nov., transferred from *Bavirecta*

*Padillothorax mulu* Maddison, sp. nov.

*Padillothorax semiostrinus* Simon, 1901

*Padillothorax taprobanicus* Simon, 1902

###### Notes.

The synonymy of *Bavirecta* with *Padillothorax* can be established now that the identity of the type species of the latter has been clarified (see below). The synapomorphies uniting them include the position of macrosetae on the first femur, flattened carapace, placement of fovea, pale thoracic “window” (Kanesharatnam and Benjamin 2018), and palp form. The deep genetic divide seen in Fig. 3 between *P. flavopunctus* and the *P. badut* species group (*P. badut* and *P. mulu*) might permit us to divide this into more than one genus, but this is unnecessary and not yet justified. The known *Padillothorax* species together make a recognizable genus, holding together well through their synapomorphies. In addition, there is no known data guiding us as to how to divide them and maintain monophyly, as we see no strong evidence resolving the trichotomy (*P. semiostrinus*, *P. flavopunctus*, *P. badut* + *P. mulu*).

###### Diagnosis.

Distinctive for the macroseta(e) in the middle of the front surface of the first leg femur, the palp with narrow distally-pointing embolus, the pale trapezoidal “window” dorsally on the thorax (Kanesharatnam and Benjamin 2018), and the flat carapace with fovea well posterior to the eyes. The first femur’s prolateral macrosetae, either one or two, are present in both males and females (Figs 32, 33). While salticids usually have macrosetae dorsally or on the front surface distally (Figs 29–31, 34, 35), the more central location (more ventral and more basal, Figs 32, 33) of *Padillothorax* is unusual, known from only a few other genera (*Epocilla*, *Padilla*, some marpissines). The thoracic window is also different from the pale area on the top of the thorax seen in other baviines (e.g., *Bavia nessagyna* in alcohol), where it is neither as pale nor does it extend so far posteriorly as in *Padillothorax*. *Stagetillus* spp., and *Maripanthus draconis* have a pale thorax, but it is not framed by dark, as most of the thorax is pale. Palp is also distinctively simple: embolus narrow, pointing distally (i.e., appears erect in standard ventral view), fused to the tegulum, with no tegular fold covering its base as in *Bavia* and many *Indopadilla*. Epigynal openings anterior with simple copulatory ducts proceeding posteriorly. Abdomen with distinct transverse bands. First legs distinctly the longest. Male endite with lobe or sharp corner, unlike *Indopadilla* (whose endite is rounded, without lobes or corners).

##### Padillothorax semiostrinus

Taxon classificationAnimaliaAraneaeSalticidae

Simon, 1901

7326FCF4-59CC-502E-8404-2FC62FDEA7C6

Figs 9 , 17 , 24 , 32 , 158–167

Padillothorax semiostrinus Simon, 1901: 71.

###### Notes.

There has been confusion about the identity of *P. semiostrinus*. Prószyński (1987 p. 105) illustrated as *P. semiostrinus* a male and female from Simon’s collection that clearly belong to the group here called *Indopadilla*, as Prószyński’s illustrations show the group’s typical diagnostic features. If his illustrations had shown *P. semiostrinus*, then *Indopadilla* would have been junior synonym of *Padillothorax*. However, Prószyński’s illustrations are misidentified, representing a species quite distinct from *P. semiostrinus*, and which we describe above as *Indopadilla vimedaba*. Simon’s (1901a, b) descriptions are sufficient to show the distinctions. He notes the thorax of *P. semiostrinus* almost twice as long as the ocular quadrangle (approx. equal in Prószyński’s drawings and in *Indopadilla* generally), the carapace flatter than *Bavia* (as high or higher in *Indopadilla*), the fovea well back of the posterior eyes (immediately behind in *Indopadilla*), the retromarginal cheliceral teeth on a conical elevation (*Indopadilla* without elevation), and the male endite with a corner (well rounded in male *Indopadilla*). The last two distinctions are clear also in Simon’s illustration (1901b, p. 461), which shows the teeth on a mound and the lateral margin of the chelicera simple, in contrast to Prószyński’s illustration which shows the teeth not on a mound but spread along a ridge, and the lateral margin of the chelicera with an extended ridge (as in *Indopadilla*). Even Simon’s name, *Padillothorax*, emphasizes the similarity to *Padilla*, whose distinctive carapace (flat, long, with short ocular quadrangle) is quite unlike the more standard carapace shown in Prószyński’s drawings and *Indopadilla* in general. Thus, Prószyński’s drawings are misidentified. He was likely misled by the vial's label, which appears much like those of Simon’s other types.

As to what is *Padillothorax semiostrinus*, we have not been able to examine the type specimens, as they have not yet been located in the MNHN Paris. However, specimens found recently in Singapore and Taiwan match well Simon’s (1901a) description, which we translate here to English (with the assistance of Charmaine Gorrie and Anna Bazzicalupo):

"♂. Length 7.5 mm. Cephalothorax low, long and oval, red-brown, darker towards the border; texture very wormy-coriaceous except for the middle of the thoracic part which is smoother. Cephalic area in front and at both sides, [and?] near the eyes, decorated with white-silver hairs. Two wide medial thoracic bands, nearly contiguous; a thin marginal line decorated with white-silver hairs. Few white hairs around the eyes. Clypeus very narrow, bald. Abdomen narrow and very long, decorated above with dark violet, a medial band that is wide, entire, and yellow-brick red, bordered in front with lines and behind with a series of spots covered with silver-white hairs, marked on each side with a straight line in front and two white oblique [or crosswise?] lines behind. Venter reddish-yellow. Spinnerets dark. Chelicerae shiny black, short and diverging, convex outside, inside somewhat ribbed, inferior margin having a sunken furrow, then very raised and armed with a series of contiguous teeth, the middle larger. Mouth area black. Laminae truncate at the tip, convex, but with a compressed corner that is slightly extended. Sternum yellow. First pair of legs much longer and thicker than the others, femur clavate, tibia long and ovate, dark brown, coxa and femur black, tarsus yellow, tibia and metatarsus with fringe of reasonably long but not very dense black hairs. Remaining legs pale yellow, armed by a few small spines, as in *Bavia*. Palps reasonably small, pale yellow, thick with white hairs; tibia and patella rather short, equipped at the outside with a [long?] apophysis with a straight front and a black and acute tip."

**Figures 158–167.**
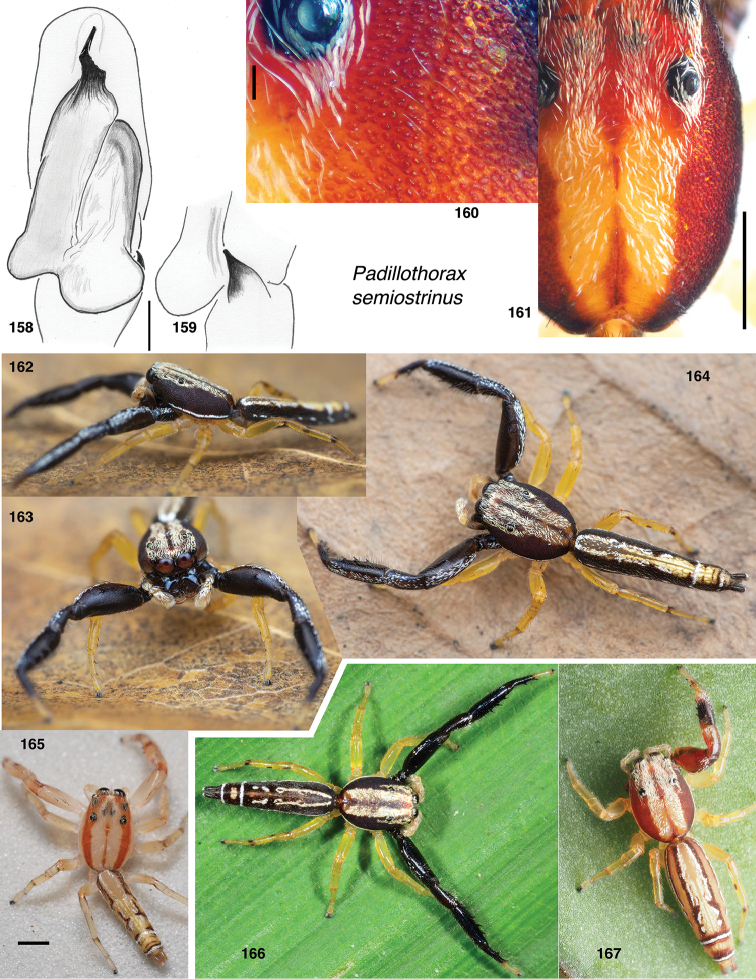
*Padillothorax semiostrinus***158**–**164** male JK.20.06.20.001 from Singapore **158** male left palp, ventral view **159** same, retrolateral view of tibia **160** oblique dorsal-lateral view just below posterior eye, showing sculpturing **161** carapace, dorsal view **162–164** living male (photographs Yongi Ng 2020) **165** juvenile (specimen AS19.2448, Singapore) **166** male (from Taichung, Taiwan, photograph Liu Shu Fen 2020) **167** female (from Taichung, Taiwan, photograph Otto Lee 2020). Scale bars: 0.1 mm (**158–160**); 1.0 mm (**161, 165**).

There are two apparent or possible conflicts between this description and the specimen seen in Figs 158–164. The most serious conflict is that the RTA in Fig. 158 is short, but Simon’s description suggests the RTA is long. However, we were unable to understand the apparent use of genitive “*apophysi*” in “*tibia patella breviore, extus ad apicem apophysi sat longa, antice directa, apice nigra et acuta, instructa*”. What exactly is long: the tip of the apophysis, the apophysis, or the outer edge of the tibia to the tip of the apophysis? Second, his description refers to marks at the back of the abdomen that are “*obliquus*”, which would differ from Figs 164, 166 if it were translated as “oblique”, but match if translated as “crosswise”. Otherwise Simon's description and his mouthparts illustration are an excellent match to the Singapore specimen, including the distinctive pair of nearly-touching wide thoracic bands, the pattern of the abdomen, the thorax rugosity except in the middle, the shape of the carapace and position of the fovea, the shininess and shape of the chelicerae, the mound bearing the retromarginal cheliceral teeth, the shape of the endite, the colours of the legs, and the yellow sternum.

We therefore provisionally identify the specimen of Figs 9, 17, 24, 32, 158–164 as *P. semiostrinus*. Although we might have added “cf.” to its label to indicate our uncertainty (“P. cf. semiostrinus”), we avoid this so as to propose a stable concept of the species that could endure if Simon’s types are never found.

A juvenile found in Singapore (Fig. 165) suggests the likely appearance of females. Males and females of this or a very similar species have been found in Taiwan, not yet examined, but with photographs posted in the website Facebook (Figs 166, 167).

**Natural history**. The male in Singapore was found in the open on a simpoh air leaf (*Dillenia suffruticosa*). A video of the living juvenile (specimen AS19.2448) is available in Maddison (2020).

###### Material examined.

Adult male (specimen JK.20.06.20.001), in LKCNHM, from Singapore: Mandai Road, 1.4106°N, 103.7631°E. Y. Ng 20 June 2020. Juvenile (specimen AS19.2448), in UBCZ, from Singapore: Palau Ubin, 1.406°N, 103.971°E, 11 June 2019 Maddison/Morehouse et al. WPM#19-048.

##### Padillothorax flavopunctus

Taxon classificationAnimaliaAraneaeSalticidae

(Kanesharatnam & Benjamin, 2018) comb. nov.

0B4F64BA-B6CD-586A-8049-A3ABD3C148D7

Bavirecta flavopuncta Kanesharatnam & Benjamin, 2018: 4–8. figs 1–3.

###### Notes.

This species shares the diagnostic features of the genus, including macrosetae on the middle of the front face of the first femur. *P. flavopunctus* has two such macrosetae on each femur, as in *P. badut* and *P. mulu*, but larger and placed even more proximally. We have concerns that the paratype female (IFS_SAL_679) is not conspecific with the male holotype, but if not conspecific, it is a closely related species, both by its strong morphological similarity (body form, markings) to the male and by its molecular proximity to a juvenile (IFS_SAL_1017) collected alongside the male at the type locality.

##### Padillothorax badut

Taxon classificationAnimaliaAraneaeSalticidae

Maddison, sp. nov.

02559CFC-7863-5109-897C-275A061F9D6B

http://zoobank.org/1EA4DDD5-DE4A-456E-90AD-16C6E5B42FCF

Figs 10 , 33 , 168–175

###### Type material.

All from Malaysia: Sarawak: Lambir Hills Nat. Pk., and in UBCZ. ***Holotype*** male (specimen IDWM.20007) from Bukit Pantu Trail, 4.2035°N, 114.0304°E to 4.2039°N, 114.0303°E, 210 m el. 5 April 2012 Maddison/Piascik/Ang WPM#12-136; ***paratype*** female (specimen SWK12-4350) from Lepoh-Ridan Trail, 4.2019°N, 114.0278°E to 4.2019°N, 114.0275°E, 190 m el. 2 April 2012 Maddison/Piascik WPM#12-125; ***paratype*** male (specimen SWK12-4688) from Inoue Trail, 4.2000°N, 114.0353°E to 4.2002°N, 114.0350°E, 190 m el. 4 April 2012 Maddison/Piascik/Ang WPM#12-128; ***paratype*** female (specimen IDWM.20008) from Bukit Pantu Trail, 4.2035°N, 114.0304°E to 4.2039°N, 114.0303°E, 210 m el. 5 April 2012 Maddison/Piascik/Ang WPM#12-136.

###### Etymology.

From the Malay word *badut*, meaning clown. In the field we called these (and *P. mulu*) the “banded clowns”. *Other names*: In Maddison (2015b) and WPM’s field or lab notebooks this species was grouped with *P. mulu* under the informal name “BVBND”, until it was distinguished from that species as “BVBND-L”.

###### Diagnosis.

Very similar to *P. mulu*, differing most notably in details of genitalia. The embolus of *P. badut* lacks the prolateral basal ridges and has a longer terminal part; the epigyne has the openings hidden beneath a fold.

###### Notes.

This and the other new Malaysian species (*P. mulu*) are very similar in appearance, thin and banded; together we consider them as the *P. badut* species group. When the first legs are held forward in life (e.g., Fig. 174), the spider appears as a series of transverse white bands approximately evenly spaced from anterior to posterior: the first leg annuli, the two transverse bands on the carapace, and the four transverse bands on the abdomen. Their bodies are more delicate and parallel-sided than other *Padillothorax*, and they have more complex emboli.

**Figures 168–175.**
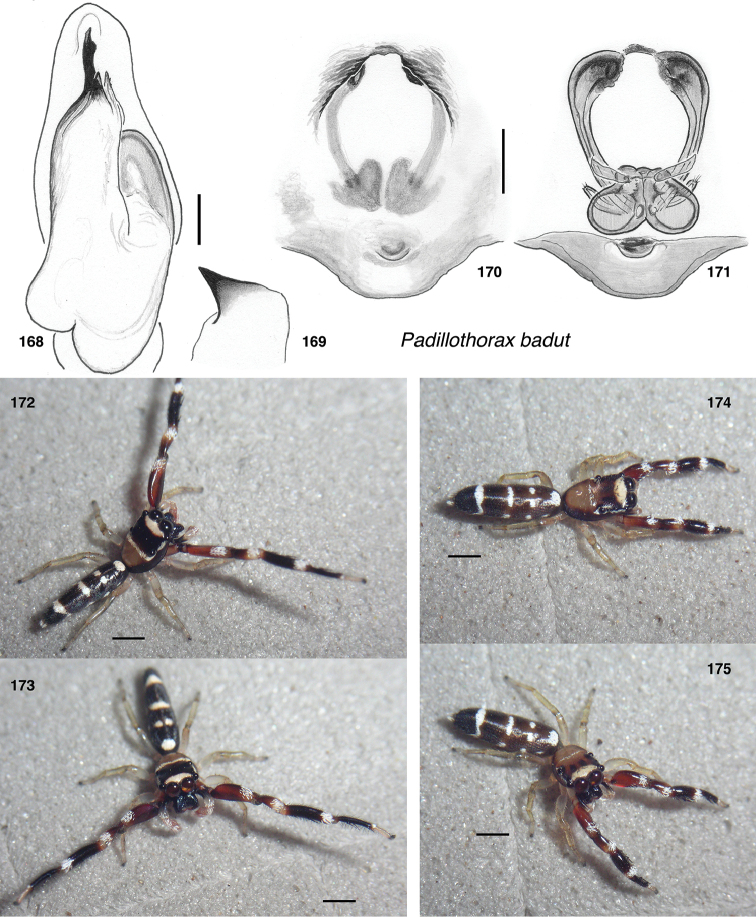
*Padillothorax badut* sp. nov. **168** male left palp, ventral view (holotype IDWM.20007) **169** same, retrolateral view of tibia **170** epigyne, ventral (specimen IDWM.20008) **171** vulva, dorsal **172, 173** Male (SWK12-4688) **174, 175** female (SWK12-4350). Scale bars: on genitalia 0.1 mm; on bodies 1.0 mm.

###### Description.

**Male** (holotype, specimen IDWM.20007). Carapace length 2.3; abdomen length 3.1. ***Carapace*** (Fig. 172): Black to dark brown except for band just back of the anterior eye row, and the pale trapezoidal window on thorax. Unusually flat (carapace height 0.72), with ocular area and most of the thorax on a plane, declining suddenly only near back of thorax. Fovea well behind the PLEs (by more than their diameter). ***Clypeus*** very narrow, dark. ***Chelicerae*** vertical, glabrous, dark. Teeth not examined in holotype, but another male from Lambir Hills has five retromarginal teeth, together in a mound as in *P. semiostrinus*. ***Palp*** pale except for base of femur. Base of embolus with two peaked projections on the retrolateral side (Fig. 168). Endite with lateral lobe (Fig. 10). ***Legs***: First legs very distinctly longest and darkest, red-brown to black except for strong annulae with white scales. Legs II–IV pale yellow with just a smudge of dark laterally on a few segments of the fourth leg. ***Abdomen***: narrow, with four white transverse bands.

**Female** (paratype, specimen IDWM.20008). Carapace length 2.1; abdomen length 3.1. Colour and structure matches that of male in nearly all aspects, with the most distinct difference being the slightly shorter first legs. **Cheliceral** teeth not examined in this specimen, but another female from Lambir Hills has four retromarginal teeth, together in a mound. ***Epigyne*** (Fig. 170) with two anterior openings, hidden under folds, and simple copulatory ducts visible without clearing. The copulatory duct has a diverticulum for the accessory gland extending laterally just before entering the simple spermatheca.

###### Natural history.

On large-leaved understory plants such as palms. In life, they often hold the front legs out or to the front.

###### Additional material examined.

All from Malaysia: Sarawak: Lambir Hills Nat. Pk., collected 4–6 April 2012 by Maddison/Piascik/Ang, in UBCZ. One female (specimen d548) from Inoue Trail, 4.2000°N, 114.0353°E to 4.2002°N, 114.0350°E, 190 m el. WPM#12-128. One male two females from Bukit Pantu Trail, 4.2028°N, 114.0305°E to 4.2032°N, 114.0305°E, 210 m el. WPM#12-134. One female from Bukit Pantu Trail, 4.2043°N, 114.0302°E to 4.2047°N, 114.0303°E, 210 m el. WPM#12-138. One female two juveniles from Bukit Pantu Trail, 4.2047°N, 114.0303°E to 4.2052°N, 114.0303°E, 200 m el. WPM#12-139. One female from Pantu Trail,4.2027°N, 114.0401°E to 4.2030°N, 114.0399°E, 150 m el. WPM#12-144.

##### Padillothorax mulu

Taxon classificationAnimaliaAraneaeSalticidae

Maddison, sp. nov.

369EA485-7E05-517B-8A64-88678B2AAD26

http://zoobank.org/3B3E7E45-51BF-40F4-BD48-2E0CB877F732

Figs 18 , 25 , 176–187

###### Type material.

All from Malaysia: Sarawak: Mulu Nat. Pk., in UBCZ. ***Holotype*** male (specimen IDWM.20009) from Summit Trail near Camp 1, 4.0496°N, 114.8589°E to 4.0493°N, 114.8594°E, 220 m el. 21 March 2012 Maddison/Piascik/Ang WPM#12-067. ***Paratype*** female (specimen SWK12-EP0105) from Summit Trail near Camp 1, 4.0489°N, 114.8606°E to 4.0486°N, 114.8610°E, 280 m el. 21 March 2012 Maddison/Piascik/Ang WPM#12-071. ***Paratype*** male (specimen SWK12-2556) from Summit Trail near Camp 1, 4.0491°N, 114.8601°E to 4.0489°N, 114.8606°E, 270 m el. 21 March 2012 Maddison/Piascik/Ang WPM#12-070. ***Paratype*** female (specimen SWK12-EP0108) and male (specimen IDWM.20010) from Base Trail near Camp 1, 4.0543°N, 114.8534°E to 4.0544°N, 114.8531°E, 130 m el. 23 March 2012 Maddison/Piascik/Ang WPM#12-084.

**Figures 176–187.**
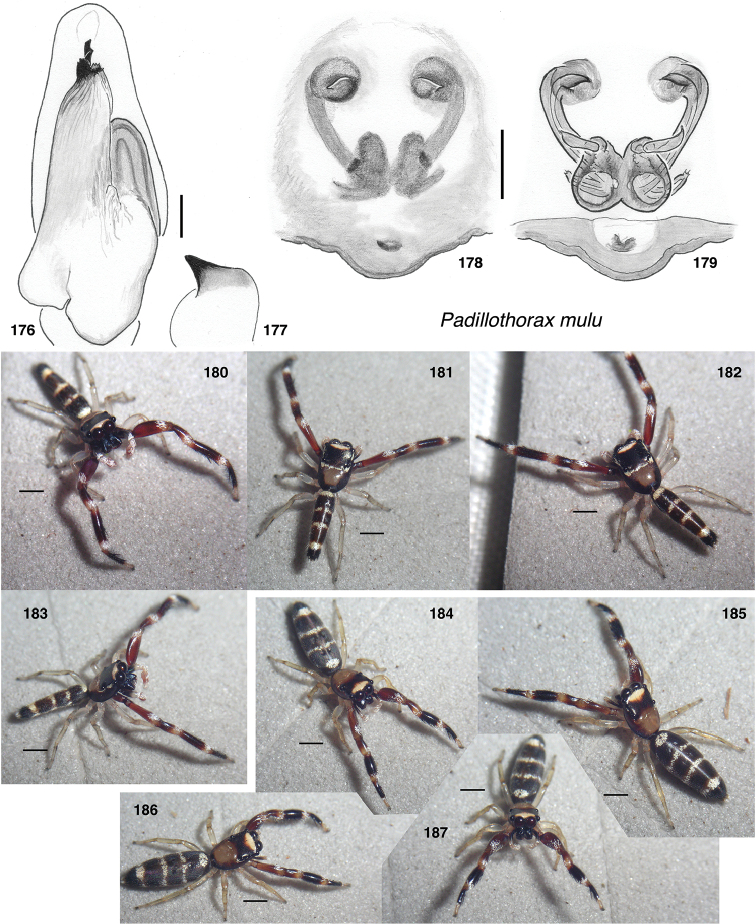
*Padillothorax mulu* sp. nov. **176** male left palp, ventral view (holotype IDWM.20009) **177** same, retrolateral view of tibia **178** epigyne, ventral (specimen SWK12-EP0105) **179** vulva, dorsal **180–183** Male (SWK-2556) **184–187** female (SWK12-2574). Scale bars: on genitalia 0.1 mm; on bodies 1.0 mm.

###### Etymology.

From the name of the type locality. *Other names*: In Maddison (2015b) and WPM’s field or lab notebooks this species was grouped with *P. badut* under the informal name “BVBND”, until it was distinguished from that species as “BVBND-M”.

###### Diagnosis.

Very similar to *P. badut*, differing most notably in details of genitalia. The embolus of *P. mulu* is shorter, and has a toothed ridge on the prolateral base; the epigyne has the openings exposed on a more or less flat surface.

###### Description.

**Male** (holotype, specimen IDWM.20009). Carapace length 2.5; abdomen length 3.5. Colour and structure matches that of *P. badut* as described above. Cheliceral teeth not examined. ***Palp*** pale except for base of femur. Base of embolus with various fine teeth; embolus shorter than in *P. badut* (Fig. 176).

**Female** (paratype, specimen SWK12-EP0105). Carapace length 2.1; abdomen length 3.3. Colour and structure matches that of *P. badut* as described above. Cheliceral teeth not examined in this specimen, but another female from Mulu has five retromarginal teeth. ***Epigyne*** (Fig. 178) similar to that of *P. badut*, except for the openings being exposed, not under a fold.

###### Natural history.

As for *P. badut*, on large-leaved understory plants. They often hold the front legs out or to the front.

###### Additional material examined.

All from Malaysia: Sarawak: Mulu Nat. Pk., in UBCZ. One female (specimen SWK12-EP0107) from Base Trail near Camp 1, 4.0543°N, 114.8534°E to 4.0544°N, 114.8531°E, 130 m el. 23 March 2012 Maddison/Piascik/Ang WPM#12-084. One female (specimen SWK12-2574) from Summit Trail near Camp 1, 4.0491°N, 114.8601°E to 4.0489°N, 114.8606°E, 270 m el. 21 March 2012 Maddison/Piascik/Ang WPM#12-070.

#### The Piranthus Clade (*Maripanthus*, *Piranthus*)

##### Maripanthus

Taxon classificationAnimaliaApialesPittosporaceae

Maddison gen. nov.

35B56345-B640-5CEE-916E-AF30E1E82139

http://zoobank.org/59175166-B0A7-4576-B294-856C7AE5FA5C

###### Type species.

*Maripanthus draconis* Maddison, sp. nov.

###### Species included.

*Maripanthus draconis* Maddison, sp. nov.

*Maripanthus jubatus* Maddison, sp. nov.

*Maripanthus menghaiensis* (Cao & Li, 2016), comb. nov. (transferred from *Nannenus*)

*Maripanthus reinholdae* Maddison, sp. nov.

*Maripanthus smedleyi* (Reimoser, 1929), comb. nov., transferred from *Bavia*

###### Etymology.

An arbitrary combination of letters, reminiscent of *Marpissa* (as the females resemble) and *Piranthus* (to which it is closely related). To be treated grammatically as masculine.

###### Diagnosis.

Epigynal atria long and gaping, anteriorly placed. Embolus long and beginning on the basal side of the tegulum, apparently freely articulated from the tegulum (as in the related *Piranthus*). Retromarginal cheliceral teeth close together, forming a single short ridge. Male endite with sharp corner (Fig. 11). Abdomen with central longitudinal pale band flanked by dark and stuttered into a chevron. Body unremarkable for the Salticinae, in contrast to its sister genus *Piranthus* which are unusually flat and robust. The small *M. menghaiensis* and *M. reinholdae* in some respects resemble *Indopadilla* or small *Bavia*, but they lack the ridged chelicera l paturon of the former and the short embolus of the latter.

##### Maripanthus draconis

Taxon classificationAnimaliaApialesPittosporaceae

Maddison, sp. nov.

8CD7BE05-252F-5372-9494-CDA75FA4DA80

http://zoobank.org/B511C74F-1343-48B8-ACDD-B01B20549324

Figs 27 , 34 , 188–201

###### Type material.

***Holotype*** male (specimen AS19.2232), two ***paratype*** females (specimens AS19.2250 and d547), all in LKCNHM, from Singapore: Bukit Timah Nature Reserve, stream at Jungle Falls Path. 1.3562°N, 103.7748°E to 1.3572°N, 103.7734°E 110–150 m elev. 12 June 2019 Maddison, Morehouse, & Marathe WPM#19-051. ***Paratype*** male (specimen WSG018) from Singapore: Nee Soon Swamp Forest. Beating vegetation. 1.39°N, 103.81°E 12 May 2005. W. Maddison, D. Li, I. Agnarsson, J. X. Zhang. WPM#05-015.

###### Etymology.

Greek, *δράκων*, referring to the fiery colours of the male. *Other names*: In Maddison (2015b) and WPM’s field or lab notebooks the informal code for this species was “CFMAR”. A specimen that is likely a closely related but distinct species is shown in Koh and Bay 2019 as “*Bavia*” sp. B Black-collared long-bellied jumping spider.

###### Diagnosis.

Most similar to *M. smedleyi*, of which only the female is known; *M. draconis* differs in having longer epigynal atria (greater than half of epigynal length) and less distinct atrial cliff (“ac”, Fig. 191). Similar to *M. jubatus* in being large bodied, but *M. draconis* differs in having a honey-coloured translucent thorax, which in the female is speckled with black, and which in the male contrasts strongly against the black ocular area. Male front is impressively red-orange, with red-orange first femora, palps, and rings around eyes, and a golden face. Embolus slightly shorter than the other three species for which males are known. Ventral bump on palp femur.

###### Description.

**Figures 188–201.**
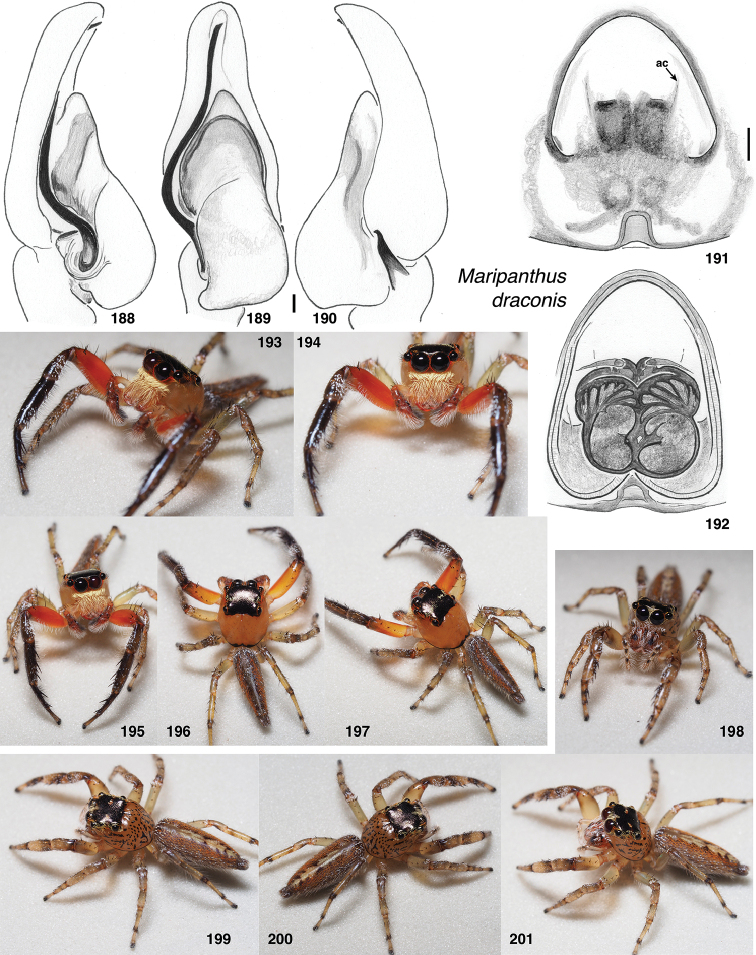
*Maripanthus draconis* sp. nov. **188** male left palp, prolateral view (holotype AS19.2232) **189** same, ventral view **190** same, retrolateral view **191** epigyne, ventral (specimen AS19.2250) **192** vulva, dorsal **193–197** holotype **198–201** female AS19.2250. Scale bars: 0.1 mm.

**Male** (based on holotype, specimen AS19.2232). Carapace length 4.0; abdomen length 5.0. ***Carapace*** (Figs 196, 197) black in ocular area and in a thin line along ventral and posterior margin; thorax strongly contrasting honey coloured, glabrous except for a few vertical black setae. ***Clypeus*** covered with yellow-cream setae. ***Chelicerae*** vertical, a soft orange-red, covered with yellow-cream setae. Three promarginal and five retromarginal teeth. ***Palp*** femur pale, darkening to red-brown distally. Bulb elongate (Figs 188, 189); embolus arising on the prolateral basal corner and proceeding distally. Femur with small but distinct distal ventral bump. Endite with small corner projection. ***Legs*** with pale femora and darker markings more distally. First femur striking orange-red, patella to tarsus dark brown to black. First patella through metatarsus with sparse ventral fringe of setae that are black at base, white at tip. ***Abdomen*** with brown longitudinal medial band dorsally, flanked by scattered red scales, with some scattered white scales basally.

**Female** (paratype, specimen AS19.2250). Carapace length 4.2; abdomen length 6.1. ***Carapace*** (Fig. 199) integument black in ocular area, except for a pale spot on each side just medial to the PME (in other females, these pale spots form a transverse band that intersects with a longitudinal medial pale band, forming a pale cross in the ocular area). Thorax honey-coloured with distinct black speckles. ***Clypeus*** with scattered white setae. ***Chelicerae*** brown with darker patches, with scattered white setae. Three promarginal and five retromarginal teeth. ***Legs*** pale honey-coloured with various darker patches. ***Abdomen*** with central longitudinal pale chevroned band flanked by red scales. ***Epigyne*** with large atria leading to broad ducts (Figs 191, 192). Atria shallow laterally, declining rapidly at an “atrial cliff” (“ac”, Fig. 191) medial to which the surface is distinctly deeper. The ECP is apparently medial and small.

###### Natural history.

In Singapore, beating vegetation in forest understory.

###### Additional material examined.

In UBCZ. Singapore: Bukit Timah Nature Reserve, stream at Jungle Falls Path. 1.3562°N, 103.7748°E to 1.3572°N, 103.7734°E, 110–150 m elev. 12 June 2019 Maddison, Morehouse, & Marathe WPM#19-051 (one female); Bukit Timah Nature Reserve. 1.355°N, 103.78°E, 29 May 2005. W. Maddison, D. Li, I. Agnarsson, J. X. Zhang. WPM#05-041 (1 female 1 juvenile). Malaysia: Johor: Gunung Belumut Recreational Forest. 2.066°N, 103.527°E, 60–100 m el. 24 May 2005. W. Maddison, D. Li, I. Agnarsson, J. X. Zhang. WPM#05-038 (1 male, 1 female, 6 juveniles); near Kluang, Gunung Lambak. 2.025°N, 103.344°E, 50–100 m el. 25 May 2005. W. Maddison, D. Li, I. Agnarsson, J. X. Zhang. WPM#05-039 (1 male). Pahang: Tanah Rata. Jungle Trail 9 from Robinson Falls. 4.46°N, 101.40°E, 1200–1500 m el. 21–22 May 2005. W. Maddison, D. Li, I. Agnarsson, J. X. Zhang. WPM#05-035 (1 female). Selangor: Ulu Gombak Field Station, 3.325°N, 101.753°E, 250 m el. 16–19 May 2005. W. Maddison, D. Li, I. Agnarsson, J. X. Zhang. WPM#05-026 (1 female 1 juvenile); canyon near Ulu Gombak, 3.325°N, 101.765°E, 275 m el. 17 May 2005. W. Maddison, D. Li, I. Agnarsson, J. X. Zhang. WPM#05-027 (1 female 1 juvenile). Sarawak: Fairy Caves, near Kuching, 1.381–2°N, 110.117–9°E, 20 m el. 10 March 2012 Maddison/Piascik/Ang/Lee WPM#12-011 (1 female). The female from Sarawak is listed with some hesitation. It may be conspecific with a male *Maripanthus* from Brunei, which appears to be a closely related but distinct species, with embolus initially directed distinctly more to the dorsal, and slight different carapace markings (specimen JK 08.08.23.0004, in LKCNHM, from Brunei: Ulu Temburong National Park, Ashton Trail 4.5428°N, 115.1528°E J K H Koh 23 August 2008).

##### Maripanthus jubatus

Taxon classificationAnimaliaApialesPittosporaceae

Maddison, sp. nov.

1BB70135-1921-5144-8F1E-0224D5C19522

http://zoobank.org/BB40722F-B763-4501-B9E6-50F9C02EF23B

Figs 11 , 202–214

###### Type material.

***Holotype*** male (specimen NCBS-BN352, also known as AS19.4373) and ***paratype*** female (specimen NCBS-BN353, also known as AS19.4996), in NCBS collection, from India: Karnataka: Kodagu: Yavakapadi, Honey Valley area, buildings and roadside, 12.22°N, 75.66°E, 1100 m elev. 23–28 June 2019 W. Maddison & K. Marathe WPM#19-069.

###### Etymology.

Latin, meaning maned or crested, referring to the field of short black setae on the male ocular area. *Other names*: In WPM’s field or lab notebooks the informal code for this species was “CFMA2”.

**Figures 202–214.**
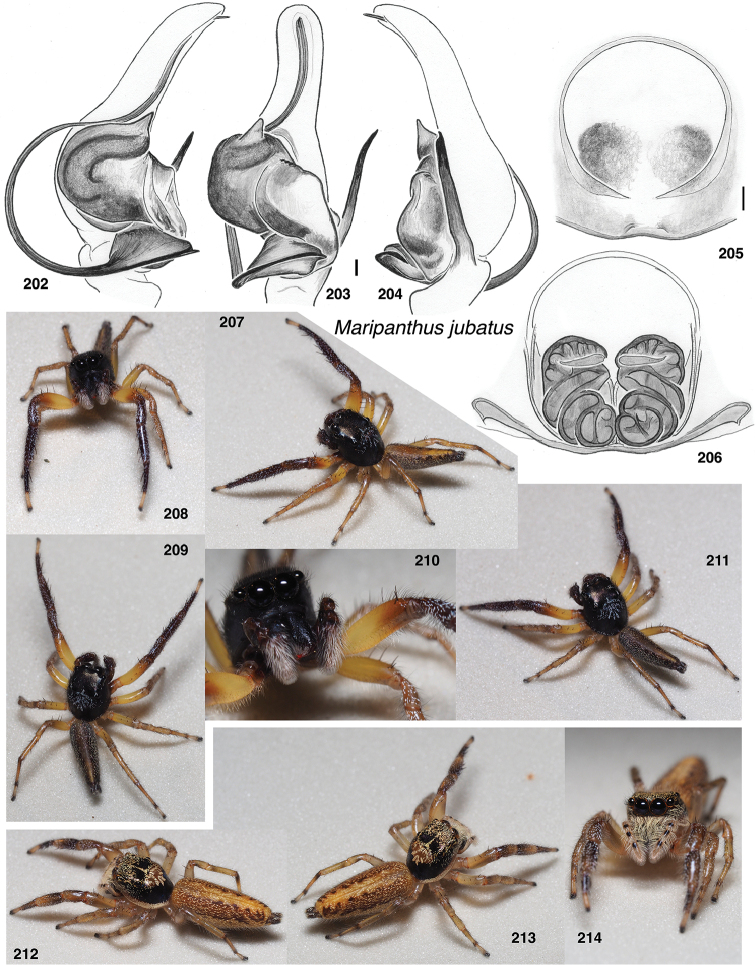
*Maripanthus jubatus* sp. nov. **202** male left palp, prolateral view (holotype NCBS-BN352) **203** same, ventral view **204** same, retrolateral view **205** epigyne, ventral (specimen NCBS-BN353) **206** vulva, dorsal **207** holotype **208–211** male NCBS-BN354 **212–214** female NCBS-BN353. Scale bars: 0.1 mm.

###### Diagnosis.

Similar size and body form to *M. draconis*, but differs most notably in solid dark integument of the carapace and face. Male palp with dramatically long embolus and RTA.

###### Description.

**Male** (based on holotype, NCBS-BN352). Carapace length 3.9; abdomen length 4.8. ***Carapace*** (Figs 207–211) black, with scattered white scales on the top of the thorax. Front half of ocular area covered with vertical black setae, giving the appearance of a mane from in front (Fig. 210). ***Clypeus*** black, with long black setae. ***Chelicerae*** vertical, black. At least five retromarginal teeth. ***Palp*** black, but cymbium with white setae. Embolus long and swooping (Figs 202–204), beginning with a broad base over the tibia, proceeding prolaterally, then dorsally, then distally. RTA long and more or less straight. Femur lacks the ventral bump seen in *M. draconis*. Endite with small corner projection. ***Legs*** similar to those of *M. draconis*, with some segments of first legs dark, but differs in having the first femur pale honey darkening terminally to brown, and tarsus pale. ***Abdomen*** light brown mid-dorsally, darker laterally and with scattered cream coloured scales.

**Female** (based on paratype, NCBS-BN353). Carapace length 4.2; abdomen length 5.1. ***Carapace*** black except medium brown areas (yellow in alcohol) around fovea and along margin, covered with yellow cream scales in band along ventral margin, and dorsally on ocular area and anterior part of thorax (Figs 212–214). Clypeus covered with long cream-coloured setae. ***Chelicerae*** dark, covered with long cream setae. Three promarginal and five retromarginal teeth. ***Legs*** pale honey-coloured with various darker patches. ***Abdomen*** like that of male, but paler (Fig. 213). ***Epigyne*** (Fig. 205) similar to that of *M. draconis*, but with ECP split into a pocket on each side.

###### Natural history.

Found in dry hanging banana leaves.

###### Additional material examined.

All in NCBS collection. One male (specimen NCBS-BN354, also known as AS19.4403) from India: Karnataka: Kodagu: Yavakapadi, on top of car, 12.2408°N, 75.6547°E, 23 June 2019 K. Marathe WPM#19-068. One male one female (specimens NCBS-BN355 and NCBS-BN355) from India: Karnataka: Kodagu: near Madikeri, Rainforest Retreat, banana plantation, 12.480°N, 75.709°E, 30 June 2019 K. Marathe WPM#19-103.

##### Maripanthus menghaiensis

Taxon classificationAnimaliaApialesPittosporaceae

(Cao & Li, 2016) comb. nov.

40A13B6F-BF69-5531-8487-09E8C2C541C1

Figs 223 , 224

Nannenus menghaiensis Cao & Li, 2016: 82–85, figs 28–29.

###### Note.

*Nannenus menghaiensis* is here transferred to *Maripanthus* (and thus to the Baviini) based on its many close similarities with *M. reinholdae*, which itself is placed in *Maripanthus* by both morphological and molecular data. *M. menghaiensis* has an elongate body and pattern of thoracic and abdominal markings very much like those of other baviines (and unlike *Nannenus*, which is a compact-bodied ground dweller). See Diagnosis of *M. reinholdae* for distinctions therefrom.

A male (specimen IDWM.20013) that is either *M. menghaiensis* or a very closely related species is shown in Figs 223 and 224 (in UBCZ, from Malaysia: Selangor: Ulu Gombak Field Station, 3.325°N, 101.753°E, 250 m el. 16–19 May 2005. W. Maddison, D. Li, I. Agnarsson, J. X. Zhang. WPM#05-026). Its palp is very much like that figured by Cao, Li and Żabka (2016), but it differs slightly in the base of the embolus and a narrower embolus.

**Figures 215–224.**
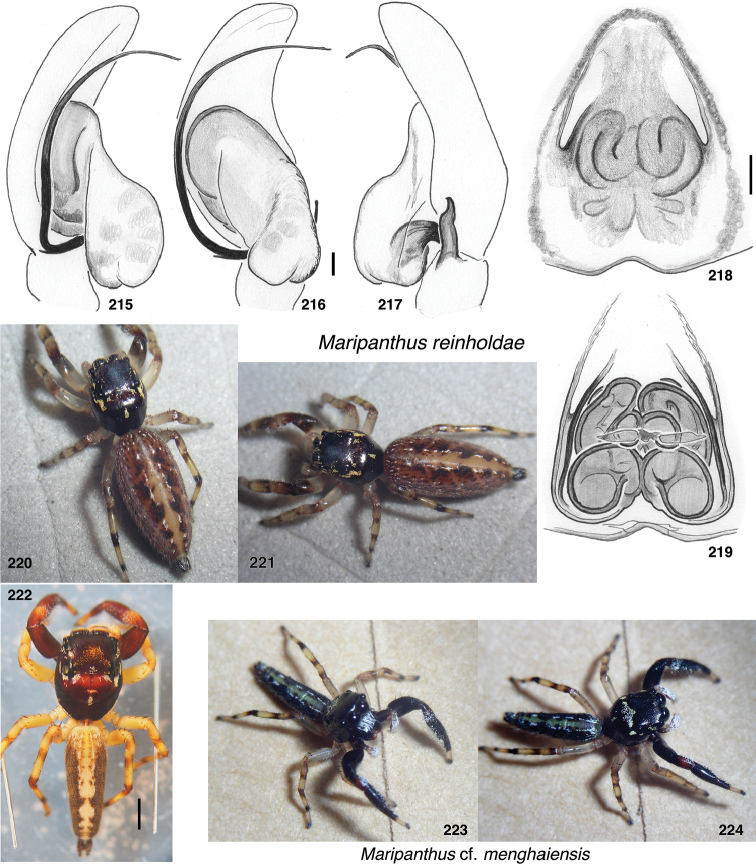
*Maripanthus reinholdae* sp. nov. and *M.* cf. *menghaiensis***215–222***Maripanthus reinholdae***215** male left palp, prolateral view (holotype JK.11.12.24.0006) **216** same, ventral view **217** same, retrolateral view **218** epigyne, ventral (specimen SWK12-1934) **219** vulva, dorsal **220, 221** female SWK12-1934 **222** holotype male **223, 224** male *M.* cf. *menghaiensis* (specimen IDWM.20013). Scale bars on genitalia 0.1 mm; on body 1.0 mm.

##### Maripanthus reinholdae

Taxon classificationAnimaliaApialesPittosporaceae

Maddison, sp. nov.

F09C8524-C1A1-506C-9730-F94F97B92D4D

http://zoobank.org/5B8BB349-2D63-42E2-AD0A-4426B4BAD0D9

Figs 19 , 26 , 215–222

###### Type material.

***Holotype*** male (specimen JK 11.12.24.0006), in LKCNHM, from Brunei: Ulu Temburong National Park, Canopy Walk Trail, 4.5522°N, 115.1578°E, J. K. H. Koh 24 Dec. 2011. ***Paratype*** female (specimen SWK12-1934, in UBCZ), from Malaysia: Sarawak: Mulu Nat. Pk., Botanical Trail, 4.0406°N, 114.8170°E to 4.0404°N, 114.8176°E, 50 m el. 16 March 2012 Maddison/Piascik/Ang WPM#12-044. ***Paratype*** female (specimen SWK12-1991, in UBCZ), also from Mulu Nat. Pk., Kenyalang Trail, 4.0229°N, 114.8128°E to 4.0228°N, 114.8134°E, 55 m el. 17 March 2012 Maddison/Piascik/Ang WPM#12-047.

###### Etymology.

Named in honour of Christa Deeleman-Reinhold, whose extensive work on southeast Asian spiders has greatly increased our knowledge of the area’s fauna. She has discovered and described over 350 new species, including 54 new salticids. *Other names*: In Maddison (2015b) and WPM’s field or lab notebooks the informal code for this species was “BVBGB”. This species is shown in Koh and Bay 2019 as “*Bavia*” sp. C Two-lined long-bellied jumping spider; the upper photograph on p. 208 is of the holotype.

###### Diagnosis.

Very similar to *M. menghaiensis*, and like it smaller and more *Indopadilla*-like in body form than *M. draconis* and *M. jubatus* (shorter first legs, more elongate abdomen). Differs from *M. menghaiensis* in the longer epigynal openings, and in details of the palp’s bulb. In view from the retrolateral, the embolus is first directed to the distal then quickly turns dorsally (*M. menghaiensis*, embolus begins toward the dorsal). When the embolus comes out from behind the tegulum it is directed slightly proximally (slightly distally in *M. menghaiensis*). The embolus is thinner near the tip than in *M. menghaiensis*.

###### Description.

**Male** (based on holotype, specimen JK.11.12.24.0006; living holotype shown on p. 208 of Koh and Bay 2019). Carapace length 2.9; abdomen length 4.0. ***Carapace*** (Fig. 222) black except for brown around fovea. Cream scales form a band crossing the thorax behind the PLEs, a small streak on the midline of the thoracic slope, and a narrow band along the lateral margins. ***Clypeus*** dark but centrally with a cluster of erect white scales that overhang the chelicerae. ***Chelicerae*** simple, vertical, dark. Plurident. ***Palp*** pale except for dark femur. Embolus long, arising retrolaterally before curling under the bulb prolaterally then proceeding distally (Figs 215–217). A short spur diverges from the embolus before it turns distally (Figs 215, 216). RTA vertical except for a bend and curl distally. Endite with small sharp corner, similar but smaller to that in the larger species of *Maripanthus*. ***Legs*** pale honey coloured except for first, which is black in all segments except the tarsus. Third and fourth legs darker near more distal joints. ***Abdomen*** thin, dark, with a pale mid-dorsal band, just lateral to which are indistinct longitudinal streaks of white scales.

**Female** (based on paratype, specimen SWK12-1934). Carapace length 3.7; abdomen length 5.0. ***Carapace*** as in male, but slightly paler in integument. ***Chelicerae*** with three promarginal and five retromarginal teeth. ***Legs*** as in male but with first legs only slightly darker than the others. ***Abdomen*** brown with central pale chevroned band, and thin white streaks as in male. ***Epigyne*** (Fig. 218) similar in conformation to *M. draconis* and *M. jubatus*, but with atria smaller and copulatory ducts not so compacted.

###### Additional material examined.

One female (specimen JK.12.02.04.0010, in LKCNHM) from Brunei: Belait, Trail To Wasai Teraja Secondary Forest, 4.2911°N, 114.4231°E, J. K. H. Koh 4 February 2012.

##### Maripanthus smedleyi

Taxon classificationAnimaliaApialesPittosporaceae

(Reimoser, 1929) comb. nov.

1A730F71-4A0D-5E71-9515-EFED59B3EE42

Figs 225–228

Bavia smedleyi Reimoser, 1929: 130–132, fig. 4, holotype female SMF 1127 in SMF from Siberut, Sumatra, examined.

###### Notes.

**Figures 225–228.**
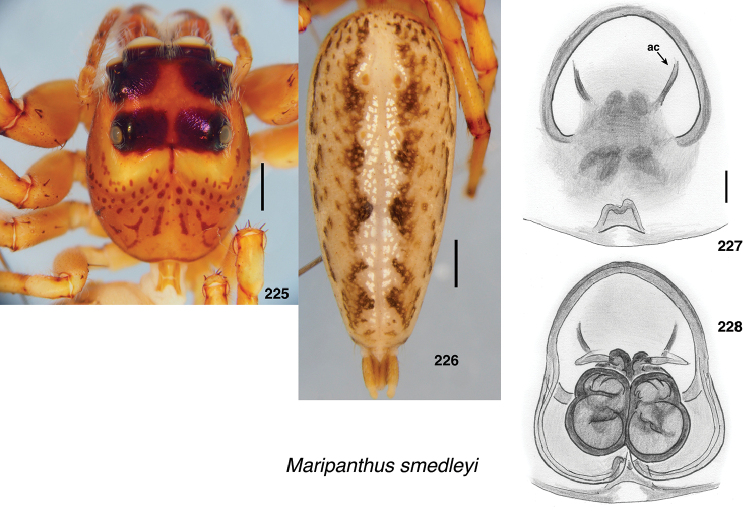
*Maripanthus smedleyi*, female holotype (SMF 1127) **225** carapace **226** abdomen **227** epigyne, ventral **228** vulva, dorsal. Scale bars: on genitalia 0.1 mm; on body 1.0 mm.

This species is close to *M. draconis*, with similar body form and markings (Figs 225, 226), but differing in details of the epigyne (Figs 227, 228). The atria are shorter, ~ ½ length of the epigyne. Within the atria, the central depressed area is separated from the lateral raised areas by a more distinct and more heavily sclerotized “atrial cliff” (“ac”, Fig. 227). The spermathecae are smaller.

##### Piranthus

Taxon classificationAnimaliaAraneaeSalticidae

Thorell, 1895

78155BB3-24C7-5319-854B-94E943E074E5

Piranthus Thorell, 1895. Type species *Piranthus decorus* Thorell, 1895.

###### Species included.

*Piranthus api* Maddison, sp. nov.

*Piranthus bakau* Maddison, sp. nov.

*Piranthus decorus* Thorell, 1895

*Piranthus kohi* Maddison, sp. nov.

*Piranthus mandai* Maddison, sp. nov.

*Piranthus planolancis* Malamel, Nafin, Sudhikumar & Sebastian, 2019

###### Diagnosis.

Carapace surface rugose, with a coarse reticulate sculpturing throughout. Carapace flat (height well less than half the length), with ocular area and front part of thorax on a plane, and fovea well back of PLE, 1.3–1.5 × further from front of carapace than is the back of the PLE. Legs robust, especially the first pair. Embolus begins at prolateral basal corner of bulb; epigyne with central septum. Tip of abdomen black.

Two of the species (*P. bakau* and *P. kohi*) are distinctive for their black-and-white banding, three others (*P. decorus*, *P. mandai*, and *P. planolancis*) are more simply marked with brown and black, while the last (*P. api*) is a red-orange-black ember.

The four new species described here extend the range of *Piranthus* eastward as far as Borneo. The two previously described species, *P. decorus* (Thorell 1895; Caleb and Sanap 2017) and *P. planolancis* (Malamel et al. 2019; Nafin et al. 2020), are from Myanmar and India. A video of a living female of *P. planolancis* (specimen AS19.5940) is available in Maddison (2020).

##### Piranthus api

Taxon classificationAnimaliaAraneaeSalticidae

Maddison, sp. nov.

22EA86F1-3A34-55AE-9237-238A8C69D722

http://zoobank.org/4ABD7844-3008-4206-8EA2-20FE15058E44

Figs 229–236

###### Type material.

***Holotype*** female (specimen AS19.3205), in LKCNHM, from Singapore: Sungei Buloh Wetland Reserve, near Visitor Centre, 1.440°N, 103.734°E, 19 June 2019 Maddison/Marathe/Morehouse/et al. WPM#19-064.

###### Etymology.

From the Malay word, “api”, meaning “fire”, referring to the colour. *Other names*: In WPM’s field or lab notebooks the informal code for this species was “PIORG”.

###### Diagnosis.

A distinctively narrow species with bright red-orange legs.

###### Description.

**Figures 229–236.**
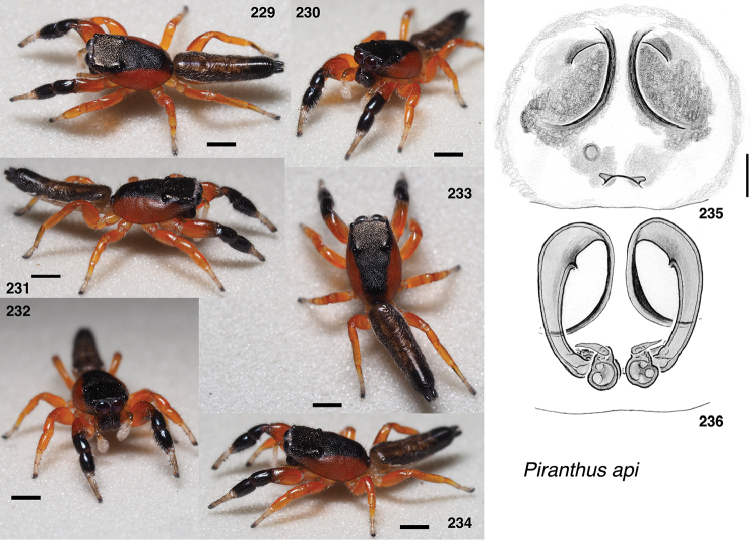
*Piranthus api* sp. nov., female holotype (specimen AS19.3205) **229–234** body **235** epigyne, ventral **236** vulva, dorsal. Scale bars: on epigyne 0.1 mm; on body 1.0 mm.

**Female** (holotype, specimen AS19.3205). Carapace length 3.0; abdomen length 3.5. ***Carapace*** narrow and low, orange-red-brown with central black area covering ocular area and medial part of thorax (Figs 229, 233). ***Clypeus*** black. ***Chelicerae*** short and vertical, dark. ***Legs*** red-orange except for black patella and tibia of first legs. ***Abdomen*** dark brown above, with median longitudinal paler band that in alcohol appears as to uneven parallel cream bands, extending backward until the black end of the abdomen. ***Epigyne*** (Fig. 235) with medial septum and a small medial ECP.

###### Natural history.

The two specimens were both found deep within large grass tussocks overhanging a moist ditch. A video of the living holotype is available in Maddison (2020).

###### Additional material examined.

A second female (specimen AS19.3217, in UBCZ), raised in captivity, same data as the holotype.

##### Piranthus bakau

Taxon classificationAnimaliaAraneaeSalticidae

Maddison, sp. nov.

08C20831-109B-547A-8FA6-CD03F9DD2518

http://zoobank.org/1273542D-3BE3-404B-870E-246EEAA12967

Figs 237–254

###### Type material.

***Holotype*** male (specimen SWK12-0561, also known as d424), in UBCZ, from Malaysia: Sarawak: Bako Nat. Pk., Mangroves, beach forest, 1.722°N, 110.446°E, 0 m el. 8 March 2012 Maddison/Piascik/Ang/Lee WPM#12-003.

###### Etymology.

Referring to the type locality and to the holotype’s habitat, mangroves (Malay, *bakau* = mangrove). *Other names*: In Maddison (2015b) and WPM’s lab notebooks the informal code for this species was “BKOMG”. This species is shown in Koh and Bay 2019 as the male of “*Bavia*” sp. D Strong-armed flat jumping spider.

**Figures 237–254.**
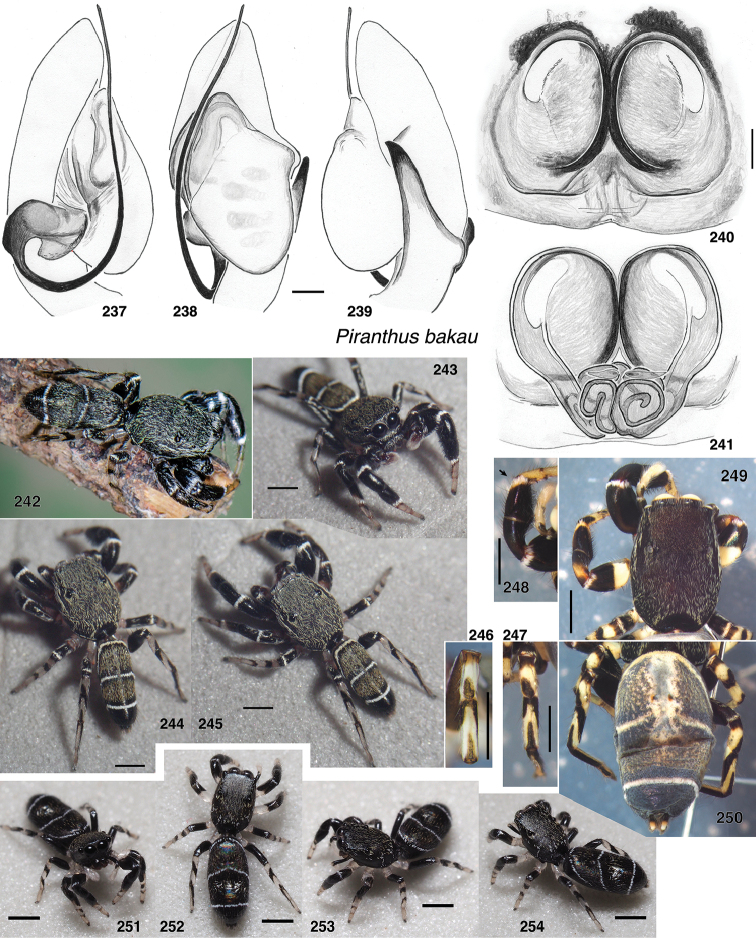
*Piranthus bakau* sp. nov. **237** male left palp, prolateral view (holotype SWK12-0561) **238** same, ventral view **239** same, retrolateral view **240** epigyne, ventral (specimen AS19.2895) **241** vulva, dorsal **242** male JK.11.04.17.0040 (photograph Joseph K. H. Koh) **243–245** male holotype (SWK12-0561) **246** male holotype left fourth patella and tibia, dorsal view (right leg digitally flipped to appear as left) **247–254** female specimen AS19.2895 **247** left fourth patella and tibia, dorsal view **248** left first leg, prolateral view **249** adult female prosoma **250** adult female abdomen **251–254** same specimen while immature. Scale bars on genitalia 0.1 mm; on bodies and legs 1.0 mm.

###### Diagnosis.

**Figures 255–269.**
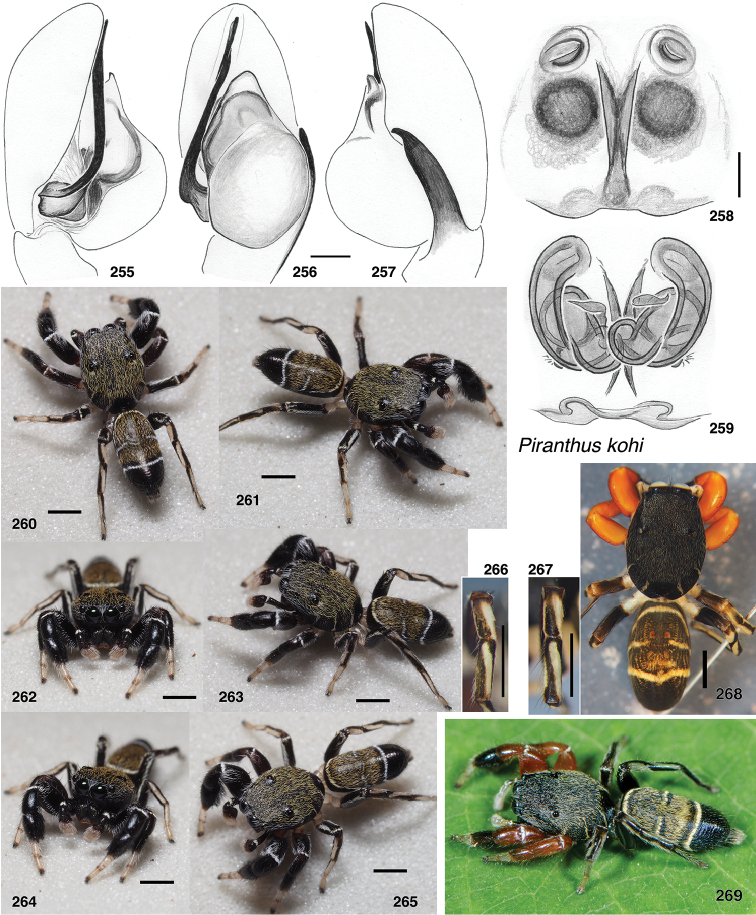
*Piranthus kohi* sp. nov. **255** male left palp, prolateral view (holotype AS19.1813) **256** same, ventral view **257** same, retrolateral view **258** epigyne, ventral (specimen JK.12.04.11.0032) **259** Vulva, dorsal **260–265** holotype **266** male holotype left fourth patella and tibia, dorsal view **267** female left fourth patella and tibia, dorsal view (specimen JK.19.07.19.0001) **268** female JK.19.07.19.0001 **269** Female JK.12.04.11.0032 (photograph Joseph K. H. Koh). Scale bars: on genitalia 0.1 mm; on bodies and legs 1.0 mm.

This and the closely similar *P. kohi* differ from other known *Piranthus* in having white transverse banding on the body and legs, and the posterior legs striped with black and translucent white. *P. bakau* (Figs 237–254) differs from *P. kohi* (Figs 255–269) in having:

- Second white transverse band on the dorsum of the abdomen (i.e., the first behind the basal band) complete or broken by only a slight space;
- Sides of thorax lacking the three distinct narrow vertical lines seen in P. kohi (at most only a hint of two);
- Carapace lateral to the PLE with a bare patch, lacking golden scales, extending from PME back to behind PLE, and lateral to the bare patch is a stripe of denser golden scales (Figs 245, 249);
- First and second tibia bicoloured (black with white tip, arrow in Fig. 248);
- Second femur bicoloured (white basally, black terminally, Fig. 249);
- Black dorsal band on the fourth tibia incomplete, beginning mid-segment and reaching to the end (Figs 246, 247).
- Carapace slightly flatter than in P. kohi.
- Embolus (Fig. 237) notably longer than in P. kohi (Fig. 255), closely resembling that of P. planolancis (Nafin et al. 2020);
- Epigyne with cavernous atria framed by a curved medial ridge, and relatively long copulatory ducts leading to a posterior tangle of tubes and spermathecae.

Juveniles have markings consistent with those of adults, and thus can be distinguished by the non-genitalic features above.

###### Description.

**Male** (based on holotype, SWK12-0561). Carapace length 2.7; abdomen length 3.0. ***Carapace*** with rugose surface, black on ocular area, dark brown otherwise, covered thinly with narrow golden to white scales except bare patch lateral to PLE, and on posterior slope. ***Clypeus*** narrow, dark, with a few white setae. ***Chelicerae*** small and vertical, dark, with a few pale setae. ***Palp*** (Figs 237–239) black except band of white scales terminally on femur, and cymbium, which is dark basally but then fades to white distally. Embolus long, arising on prolateral basal corner of bulb, looping first dorsally, then proximally, then distally. RTA a long blade. Endite margin rounded laterally. ***Legs*** relatively larger (compared to the body) than in many baviines, with first pair especially robust. First and second legs mostly dark and with terminal white annuli on the segments. First patella and tibia with ventral fringe of black setae. Third and fourth leg segments from patella to tarsus translucent white with black bands and stripes. Fourth tibia and patella with a pattern of short black and white bands. On the tibia, a black dorsal band begins not basally but half way to the end, and extends distally to the tip; basally, it is interrupted by white (Fig. 246; compare with Fig. 266). ***Abdomen*** with three narrow transverse white bands, a basal one, a second one behind that, and a third one behind that. Posterior to the third band the abdomen is a shiny black, but anterior it is dusted with golden setae.

**Female** (based on specimen AS19.2895). Carapace length 3.0; abdomen length 4.1. The specimen was collected and photographed as a small juvenile (Figs 251–254); as an adult its basic appearance is similar (Figs 249, 250). Structure and markings of carapace and legs as in male. ***Abdomen*** with second transverse band oblique. ***Epigyne*** (Fig. 240) with medial septum dividing deep broad atria. What serves as the ECP is not obvious; there may be two, folds posterior to the atrium on each side, midway between the epigastric furrow and the posteriormost part of the medial septum. If so, it may be intermediate between *P. decorus*, with a medial ECP (Caleb and Sanap 2017), and *P. planolancis* (Malamel et al. 2019; Nafin et al. 2020), with a pair of distantly separated lateral ECPs (Nafin et al. 2020: fig. 17).

###### Male-female matching.

*P. bakau* and *P. kohi* are similar in general appearance, have overlapping geographical ranges, and have been collected to date with only adult males or adult females at a locality, not both. This leads to a question of which male matches which female. Unless there are additional closely similar species in the same areas, the inferred matching is well supported by the differences in markings, carapace shape, and lengths of embolus/copulatory ducts. The male of *P. bakau* and the female from Tengkorak inferred to match it share the diagnostic traits mentioned above. Doubt might arise because of one difference in their markings: the female has the second transverse abdominal band more oblique, with its two sides meeting at a central peak, while in the holotype the band is straight across. However, the second male, from Brunei, shows a peak (Fig. 242). A juvenile co-collected with the male holotype has the band peaked, and looks very much like the female when immature. The female is not designated as a paratype, however, because of the possibility it is a different but very closely related species, given the geographical distance between it and the holotype and their different habitats.

###### Natural history.

Holotype male collected from mangroves; female from Tengkorak collected by shaking vines and understory trees near waterfall.

###### Additional material examined.

Two juveniles with same data as holotype. Also, one male (specimen JK.11.04.17.0040), in LKCNHM, from Brunei: Tutong, Tasek Merimbun, Zone C2, Palau Luba, Sungai Melunchur, 4.5817°N, 114.6872°E, J. K. H. Koh 17 Apr. 2011. One female (specimen AS19.2895), in UBCZ, from Malaysia: Johor: Gunung Belemut Forest, Lata Tengkorak, 2.055°N, 103.543°E, 250 m elev. 16 June 2019 Maddison/Morehouse/et al. WPM#19-057.

##### Piranthus kohi

Taxon classificationAnimaliaAraneaeSalticidae

Maddison, sp. nov.

8313FF76-EA09-50AD-9107-CD6B84DDCD55

http://zoobank.org/DBAC788B-2FDE-4D43-98C5-0C623368A800

Figs 255–269

###### Type material.

***Holotype*** male (specimen AS19.1813), in LKCNHM, from Singapore: Sungei Buloh Wetland Reserve, 1.440–1.447°N, 103.730–103.735°E, 10 June 2019 Maddison/Morehouse/et al. WPM#19-045. ***Paratype*** female (specimen JK 19.07.19.0001), in LKCNHM, from Singapore: Pulau Ubin, Balai Quarry Trail, 1.4178°N, 103.9850°E, leg. P. Ng 19 July 2019.

###### Etymology.

This elegant species is named in honour of Joseph Koh Kok Hong, arachnologist, conservationist, and diplomat. Koh has worked tirelessly to build peace with nature. Through his collecting and excellent books (Koh 1989; Koh and Leong 2013; Koh and Bay 2019), he has substantially promoted our knowledge of southeast Asian spiders and stimulated interest in their diversity and beauty. Koh collected the first known specimen of this species, in Brunei, and enabled our field work in Singapore during which the holotype and other specimens were collected. *Other names*: In WPM’s lab notebooks the informal code for this species was “SUBSG”. This species is shown in Koh and Bay 2019 as the female of “*Bavia*” sp. D Strong-armed flat jumping spider.

###### Diagnosis.

*P. kohi* (Figs 254–269) shares with *P. bakau* (Figs 237–254) the pattern of transverse white bands, but differs from it in having:

- Three distinct vertical lines on each side of the thorax;
- Carapace lateral to the PLE more or less uniformly covered in white to gold setae;
- Second transverse dorsal band of the abdomen well broken at the middle;
- First and second tibiae and femora solid dark, not bicoloured;
- Black dorsal stripe extends the full length of tibia 4 (Figs 266, 267);
- Shorter embolus and copulatory ducts.

Juveniles can be distinguished by the non-genitalic features above.

###### Description.

**Male** (based on holotype, AS19.1813). Carapace length 2.5; abdomen length 2.5. ***Carapace*** with rugose surface, black, dusted above with narrow golden scales. Sides and back of thorax bare except for three narrow and distinct vertical lines of pale setae. ***Clypeus*** black. ***Chelicera*** vertical and black. ***Palp*** black except for white cymbium. Embolus arising on prolateral basal corner, proceeding ventrally then curving distally (Figs 255, 256) . RTA a long blade. ***Legs*** robust, especially the first pair. First two pairs black except for tarsus and metatarsus, and white annulae terminally on femur. First patella and tibia with ventral fringe of black setae. Fourth tibia and patella with a pattern of long black and white bands. On the tibia, a black band reaches from the base (on the prolateral side) all the way to the dorsal tip, forming an oblique dorsal band (Fig. 266; compare with Fig. 246). ***Abdomen*** similar to that of *P. bakau*, with three transverse white bands, anterior to the third of which the abdomen is dusted with golden setae, posterior to it a shiny black. Second transverse white band with a broad gap in both male and female.

**Female** (based on specimen, 12.04.11.0032). Carapace length 3.4; abdomen length 4.0. Structure and markings as in male, but generally more reddish, especially first and second legs, which are red-orange-brown in the femur and patella (and tibia of the second pair). ***Epigyne*** (Fig. 258) with central septum, but lacking the large cavernous atria of other *Piranthus* species.

###### Male-female matching.

See comments under *P. bakau*. Male and female *P. kohi* share the diagnostic traits mentioned above. The matching is supported by both males and females occurring in Singapore and in similar mangrove habitats – eight specimens from Sungei Buloh including 4 males; 6 specimens from Palau Ubin including 2 females.

###### Natural history.

Specimens in Singapore were found beating trees and vines in a mangrove area. It appeared that our greatest success in finding them was when shaking woody vines. Their motion when alive has a different sense than other baviines; rather than the sharply-jumping *Indopadilla*, or the frequently waving *Padillothorax badut* group, or the more sedate *Piranthus planolancis* and *P. api*, *P. kohi* is constantly flicking up and down the first legs, palps, and abdomen, somewhat like ant mimicking salticids. A video of the living holotype is available in Maddison (2020).

###### Additional material examined.

Singapore: Sungei Buloh Wetland Reserve, 1.440–1.447°N, 103.730–103.735°E, 10 June 2019 Maddison/Morehouse/et al. WPM#19-045 (3 additional males raised in captivity, 2 juveniles, UBCZ); Sungei Buloh Wetland Reserve, Coastal Trail, 1.446°N, 103.730°E to 1.445°N, 103.735°E, 19 June 2019 Maddison, Marathe, Ng WPM#19-063 (2 juveniles, UBCZ); Pulau Ubin, Chek Jawa, 1.4122°N, 103.9908°E, 11 June 2019 Maddison, Sung, & Outomuro WPM#19-047 (1 female raised in captivity, 2 juveniles, UBCZ); Lim Chu Kang Mangroves, Tree trunks and limbs, 1.44°N, 103.70°E, 13 May 2005 W. Maddison, I. Agnarsson, J. X. Zhang. WPM#05-020 (2 juveniles, UBCZ). Malaysia: Selangor: Ulu Gombak Field Station, 3.325°N, 101.753°E, 250 m el., 16–19 May 2005 W. Maddison, D. Li, I. Agnarsson, J. X. Zhang. WPM#05-026 (1 juvenile, specimen MRB109, UBCZ). Brunei: Belait, Kuala Balai, Sungai Mendarum Damit Freshwater Swamp Forest, 4.4386°N, 114.3581°E, J. K. H. Koh 11 Apr. 2012 (1 female, specimen JK 12.04.11.0032, in LKCNHM). The specimen from Ulu Gombak, used in the molecular study, matches *P. kohi* well in markings, but it is a juvenile and thus labelled conservatively in the phylogenies as P. cf. kohi.

##### Piranthus mandai

Taxon classificationAnimaliaAraneaeSalticidae

Maddison, sp. nov.

AC8B5F37-7B3E-5789-A9BE-B16756F5F7F4

http://zoobank.org/8715C1FA-D05B-4876-9E12-BFA2EC4EB81C

Figs 270–274

###### Type material.

***Holotype*** male (specimen JK.91.05.31.0001), in LKCNHM, from Singapore: Mandai Track 15 Trail, 1.4106°N, 103.7783°E, J K H Koh 31 May 1991.

###### Etymology.

Named for the type locality. *Other names*: In lab notebooks the informal code for this species was “SGOMG”.

###### Diagnosis.

**Figures 270–274.**
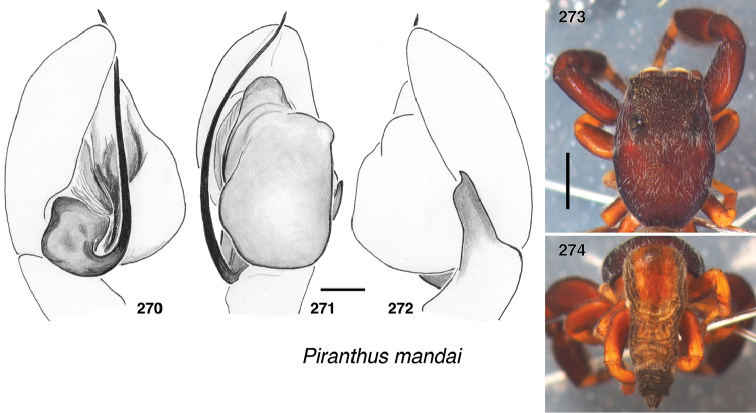
*Piranthus mandai* sp. nov., male holotype (specimen JK.91.05.31.0001) **270** left palp, prolateral view **271** same, ventral view **272** same, retrolateral view **273** prosoma **274** abdomen. Scale bars: on palp 0.1 mm; on body 1.0 mm.

In colouration similar to *P. planolancis*, browns and blacks, but with body more compact and robust, as in *P. bakau* and *P. kohi*. Embolus with only a small loop before proceeding distally, and thus *P. mandai* is second in sequence from least to most rotated embolic bases: *P. kohi* (Fig. 255), *P. mandai* (Fig. 270), *P. planolancis* (Nafin et al. 2020), *P. bakau* (Fig. 237).

###### Description.

**Male** (holotype, specimen JK.91.05.31.0001). Carapace length 2.7; abdomen length 2.5. ***Carapace*** with rugose surface, black and dark brown, with a sparse covering of narrow scales that is more or less uniform: there is no bare patch beside the PLE, and no distinct three vertical thoracic lines, but there is a slight condensation of scales into a single vertical thoracic line, similar to that in *P. bakau*. ***Clypeus*** narrow and dark. ***Chelicerae*** vertical, dark, with a few pale scales basally. ***Palp*** brown. Palp similar to that of *P. planolancis*, but RTA much shorter (Figs 270–272), the shortest known among *Piranthus*. ***Legs*** light to dark brown, with indistinct markings (Figs 273, 274) similar to those of *P. planolancis*; darkest in the femur, patella, and tibia of the first leg, and the femora of the other legs. ***Abdomen*** brown above, lacking the transverse bands of *P. bakau* and *P. kohi*, but with the dark end of the abdomen (Fig. 274).

#### Tribe Viciriini Simon, 1901

##### Nungia

Taxon classificationAnimaliaAraneaeSalticidae

Żabka, 1985

D406BF8A-CD66-56E1-8AA8-C518271EDDA6

Nungia Zabka, 1985. Type species Nungia epigynalis Żabka, 1985.

###### Note.

**Figures 275–283.**
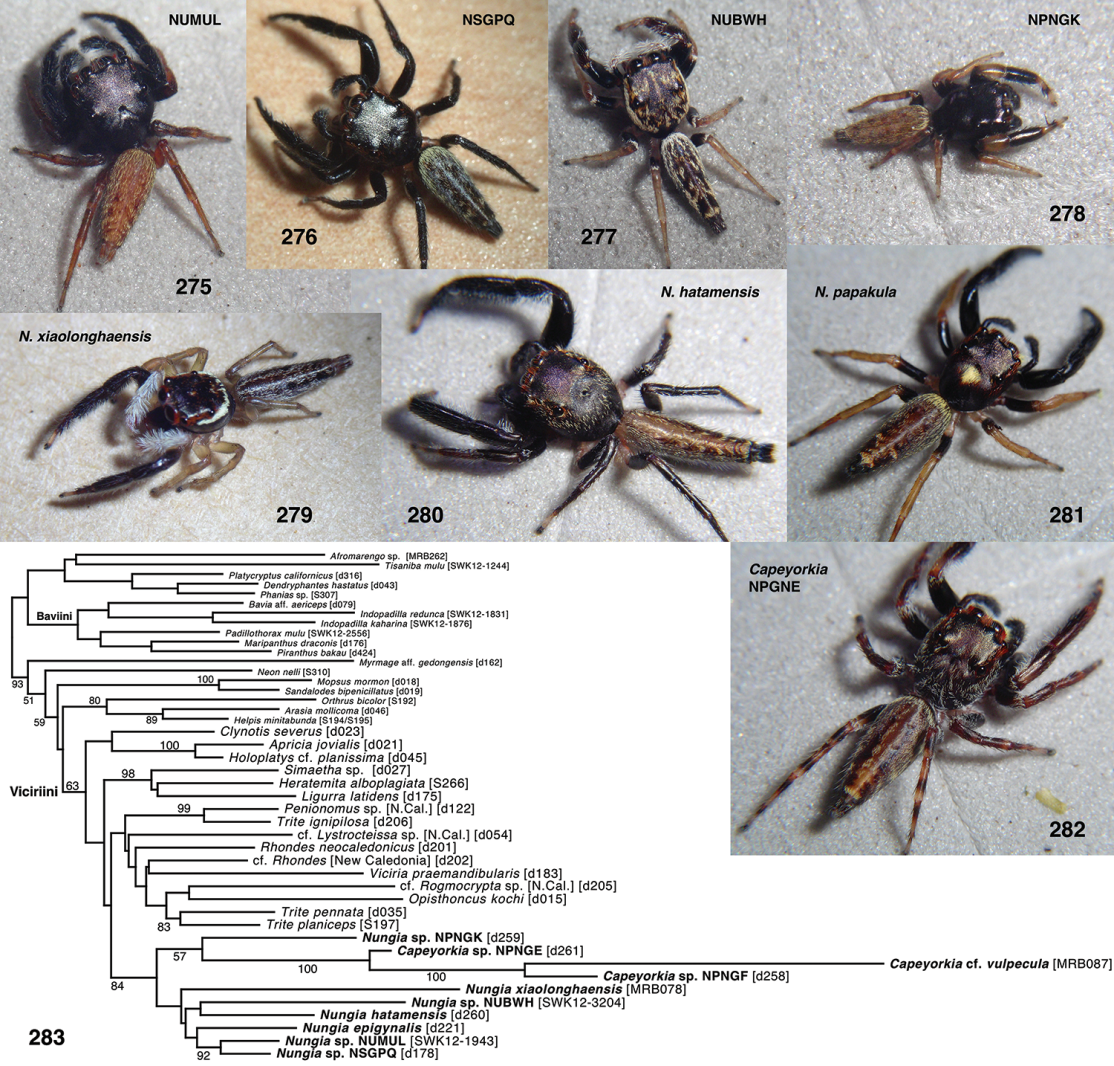
*Nungia* and *Capeyorkia* species and their phylogenetic placement, using informal species code names; all males except 277, female. **275***Nungia* sp. “NUMUL”, Sarawak (specimen SWK12-1943) **276***Nungia* sp. “NSGPQ”, Singapore (specimen d178) **277***Nungia* sp. “NUBWH”, Sarawak (specimen SWK12-3204) **278***Nungia* sp. “NPNGK”, Papua New Guinea (specimen d259) **279***Nungia xiaolonghaensis*, Pahang (specimen MRB078) **280***Nungia hatamensis*, Papua New Guinea (specimen d260) **281***Nungia papakula*, Papua New Guinea, 9.436°S, 147.364°E (specimen 2008PNG-3538) **282***Capeyorkia* sp. “NPNGE”, Papua New Guinea (specimen d261) **283** molecular phylogeny placing *Nungia* species and related (in bold) within the viciriine astioids, based on IQ-TREE maximum likelihood analysis (50 replicates, partitioned) of 28S and 16SND1 sequences, with bootstrap percentages from 500 replicates (shown only within the Astioida).

At least 16 species of elongate dull brown salticids collected in field work in Asia and New Guinea were initially thought to be baviines (example photographs, Figs 275–282), but an analysis of two genes (Fig. 283) in nine of the species show that they form a clade with the similar *Nungia epigynalis*, nested within the Viciriini. Photographs of them were released by Maddison (2015b) under the name *Nungia*. Their distinction from baviines is also morphological. They have unident or fissident chelicerae and a carapace that is dark, matte (not shiny), distinctly flat-topped, and relatively square. The male first legs are fringed below; the embolus is usually short and fixed to the tegulum. The group is unreported but diverse in New Guinea (based partly on collecting by WPM, Maddison and Zhang 2011; *Nungia* there mistakenly reported under “*Bavia* and related genera”), as might be expected for a member of the Astioida. *Capeyorkia* falls within this group (Fig. 282, 283; NPNGE and NPNGF are listed as *Capeyorkia* because of similarity of palps), and likely *Pungalina* as well. Both *Pungalina* and *Capeyorkia* may merit synonymy with *Nungia*. At least some species of *Muziris* Simon, 1901 belong here as well. Also within the group are two species misplaced in *Bavia*, one in *Diplocanthopoda*, and one in *Cosmophasis*. While we might have delayed their consideration until a future revision of *Nungia*, we address them here as part of organizing the baviines. We therefore make the following new combinations:

*Nungia hatamensis* (Thorell, 1881), comb. nov., transferred from *Diplocanthopoda* Abraham, 1925. See Fig. 280.

*Nungia modesta* (Keyserling, 1883), comb. nov., transferred from *Bavia*. Based on Keyserling’s (1883) figures, body form and colouration typical of the New Guinea *Nungia*.

*Nungia papakula* (Strand, 1911), comb. nov., transferred from *Bavia*. Male and female syntypes in SMF examined; appears closely related to *N. hatamensis* and a likely senior synonym of *Muziris wiehlei* Berland, 1938. See Fig. 281.

*Nungia xiaolonghaensis* (Cao & Li, 2016), comb. nov., transferred from *Cosmophasis* Simon, 1901. See Fig. 279.

Some of these new combinations, and others made above in baviines, correct mistaken placements that may have resulted from convergence in the general form of the male palp. The palps of *Nungia hatamensis*, *Nungia xiaolonghaensis*, *Maripanthus menghaiensis*, *Bavia capistrata*, and *Bavia maurerae* do in fact resemble those of the genera in which they had been placed, respectively, *Diplocanthopoda* (a hasariine), *Cosmophasis* (a chrysilline), *Nannenus* (a nannenine), *Evarcha* (a plexippine), and *Epidelaxia* (a nannenine). Those genera are all in the Saltafresia, a group phylogenetically distinct from the astioids and baviines. The resemblance is largely restricted to the palps, as the remainder of the body is quite distinct in each case. Convergent evolution of the basic form of the palp is widespread in salticids, insofar as the palps are simple and variation in a single dimension (embolus length) can generate palps that look superficially quite similar. The general shape of the palp should be used with caution in determining relationships.

### Species incertae sedis

The following species are too poorly known to assign to a known baviine genus, or for that matter to confirm their placement in the Baviini:

*Bavia albolineata* Peckham & Peckham, 1885

*Bavia decorata* (Thorell, 1890)

*Bavia hians* (Thorell, 1890)

*Bavia sinoamerica* Lei & Peng, 2011

Of these, *B. sinoamerica* is almost certainly not a baviine by body shape. Based on the form of the palp, and its being unident with a compact body, it may be a hasariine. *B. albolineata* has a palp that is credibly baviine, but its location in Madagascar and diverging chelicerae suggest it is not baviine.

## Supplementary Material

XML Treatment for Bavia

XML Treatment for Bavia nessagyna

XML Treatment for Indopadilla

XML Treatment for Indopadilla bamilin

XML Treatment for Indopadilla kahariana

XML Treatment for Indopadilla kodagura

XML Treatment for Indopadilla nesinor

XML Treatment for Indopadilla redunca

XML Treatment for Indopadilla redynis

XML Treatment for Indopadilla sabivia

XML Treatment for Indopadilla vimedaba

XML Treatment for Stagetillus

XML Treatment for Stagetillus opaciceps

XML Treatment for Stagetillus irri

XML Treatment for Padillothorax

XML Treatment for Padillothorax semiostrinus

XML Treatment for Padillothorax flavopunctus

XML Treatment for Padillothorax badut

XML Treatment for Padillothorax mulu

XML Treatment for Maripanthus

XML Treatment for Maripanthus draconis

XML Treatment for Maripanthus jubatus

XML Treatment for Maripanthus menghaiensis

XML Treatment for Maripanthus reinholdae

XML Treatment for Maripanthus smedleyi

XML Treatment for Piranthus

XML Treatment for Piranthus api

XML Treatment for Piranthus bakau

XML Treatment for Piranthus kohi

XML Treatment for Piranthus mandai

XML Treatment for Nungia

## Supplementary materials

Supplementary material 1 Blended spider and arachnid UCE probe set Data type UCE probes Explanation note File blending the Starrett et al. (2017) arachnid probeset and the Kulkarni et al. (2019) probeset, used to recover UCEs from contigs. The Methods section of the paper and the README file explain. https://binary.pensoft.net/file/487561This dataset is made available under the Open Database License (http://opendatacommons.org/licenses/odbl/1.0/). The Open Database License (ODbL) is a license agreement intended to allow users to freely share, modify, and use this Dataset while maintaining this same freedom for others, provided that the original source and author(s) are credited.Wayne P. Maddison, Imara Beattie, Kiran Marathe, Paul Y. C. Ng, Nilani Kanesharatnam, Suresh P. Benjamin, Krushnamegh Kunte
